# Comparative effectiveness and safety of risankizumab vs ustekinumab in patients with Crohn’s disease: a propensity score–matched multicenter retrospective cohort study

**DOI:** 10.1093/crocol/otag096

**Published:** 2026-08-18

**Authors:** Bisher Sawaf, Umberto Battistin, Mohammed Abu-Rumaileh, Kyrillos Mahrous Gerges, Yusuf Hallak, Shahem Abbarh, Elias Batikh, Nwal Hadaki, Muhammed Elhadi, Miguel Regueiro, Mohammad Al-Haddad

**Affiliations:** Internal Medicine, University of Toledo, Toledo, OH, United States; Internal Medicine, University of Toledo, Toledo, OH, United States; Division of Gastroenterology and Hepatology, Indiana University School of Medicine, Indianapolis, IN, United States; Faculty of Medicine, Sohag University, Sohag, Egypt; Internal Medicine, University of Toledo, Toledo, OH, United States; Internal Medicine, MedStar Health Georgetown University, Baltimore, MD, United States; Department of Internal Medicine, John H. Stroger, Jr. Hospital of Cook County, Chicago, IL, United States; Division of Gastroenterology and Hepatology, Indiana University School of Medicine, Indianapolis, IN, United States; Faculty of Medicine, University of Tripoli, Tripoli, Libya; College of Medicine, Korea University, Seoul, South Korea; Department of Gastroenterology, Hepatology, and Nutrition, Cleveland Clinic, Cleveland, Ohio, United States; Division of Gastroenterology and Hepatology, Indiana University School of Medicine, Indianapolis, IN, United States; Division of Gastroenterology and Hepatology, School of Medicine, The University of Jordan, Amman, Jordan

**Keywords:** Crohn’s disease, risankizumab, ustekinumab, real-world data, propensity score matching, TriNetX

## Abstract

**Background:**

In the SEQUENCE trial, risankizumab was non-inferior for clinical remission and superior for endoscopic remission compared to ustekinumab in anti-TNF-exposed Crohn’s disease (CD) patients. Given unclear results in real-world settings, we aimed to evaluate whether these findings translate to real-world advanced therapy-exposed and naïve CD cohorts.

**Methods:**

A retrospective cohort study using the TriNetX database with 1:1 propensity-score matching (PSM) analyzed 2 CD cohorts (advanced therapy-exposed and naïve) from inception to March 29, 2026. Primary outcomes were corticosteroid use, hospitalization, emergency department (ED) visits, intestinal surgery, intestinal complications, C-reactive protein (CRP) ≥ 10 mg/L, and therapy switching. Secondary outcomes included venous thromboembolism (VTE), major adverse cardiovascular events (MACE), and opportunistic infections. Odds ratios (ORs) with 95% confidence intervals (CIs) were calculated, with outcomes assessed at 12 and 24 months.

**Results:**

After PSM, each group in the advanced therapy-exposed cohort comprised 1799 patients, and each group in the advanced therapy-naïve cohort comprised 1776 patients. In the advanced therapy-exposed cohort, compared with ustekinumab, the risankizumab group had a statistically significant lower odds of ED visits, intestinal fistula, CRP ≥10 mg/L, MACE, and opportunistic infections at 12 months, while hospitalization (OR = 0.67, 95% CI, 0.50-0.91), ED visits (OR = 0.49, 95% CI, 0.35-0.68), overall intestinal surgery (OR = 0.68, 95% CI, 0.52-0.90), small intestine surgery (OR = 0.71, 95% CI, 0.52-0.99), large intestine surgery (OR = 0.72, 95% CI, 0.53-0.97), intestinal obstruction (OR = 0.69, 95% CI, 0.51-0.93), intestinal fistula (OR = 0.53, 95% CI, 0.36-0.78), CRP ≥10 mg/L (OR: 0.62, 95% CI, 0.46-0.85), MACE, and opportunistic infections at 24 months. Similarly, in the naïve cohort, compared with ustekinumab, risankizumab had a statistically significant lower odds of only CRP ≥10 mg/L at 12 months, while hospitalization, ED visits, overall intestinal surgery, small intestine surgery, large intestine surgery, anal surgery, overall complication of intestines, intestinal obstruction, switching to another advanced therapy, CRP ≥10 mg/L, and opportunistic infections at 24 months.

**Conclusions:**

In real-world practice, risankizumab was associated with modestly more favorable outcomes in patients with prior advanced therapy exposure, whereas differences became more apparent at 24 months in advanced therapy-naïve patients. Prospective studies are needed to confirm these findings.

## Graphical abstract

**Graphical Abstract:**
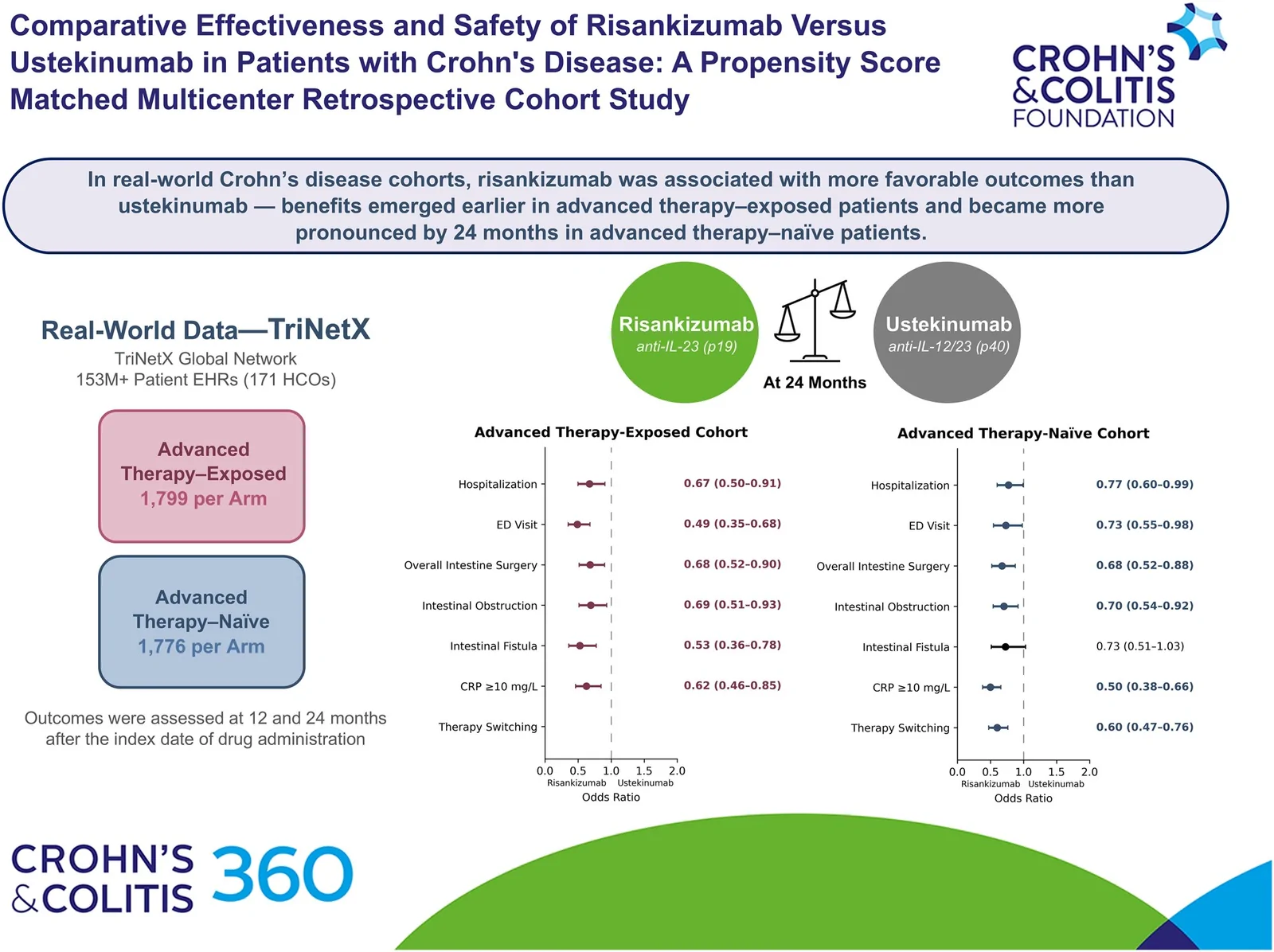

## Introduction

Inflammatory bowel diseases (IBD), including ulcerative colitis (UC) and Crohn’s disease (CD), affect approximately 0.5% of individuals in Western countries.^1^ CD is a chronic, relapsing inflammatory condition characterized by transmural inflammation that can affect any segment of the gastrointestinal tract.^2^ Advanced therapies, including biologics and small molecules, are indicated for the treatment of moderate-to-severe CD. Since the advent of anti-tumor necrosis factor-alpha (TNF-α) agents, the therapeutic armamentarium for treating has expanded to include anti-integrins, anti-interleukin 12/23 (IL-12/23), and anti-interleukin 23 (IL-23), as well as small molecules, including Janus kinase inhibitors (JAKi).^3–8^

The expanding range of advanced therapies has created uncertainty regarding optimal treatment selection and sequencing. Ustekinumab, a humanized IgG1 monoclonal antibody targeting the shared p40 subunit of IL-12 and IL-23, was approved for CD in 2016 based on demonstrated safety and efficacy in clinical trials.^5^^,^^9^^,^^10^ Most recently, risankizumab, which targets the p19 subunit of IL-23, was approved in 2022 for CD following positive registration trials.^6^^,^^7^^,^^11^^,^^12^

In a head-to-head randomized clinical trial, risankizumab outperformed ustekinumab in patients with severe plaque psoriasis, suggesting potential advantages of selective IL-23 p19 inhibition over dual IL-12/23 p40 blockade.^13^ In CD, the SEQUENCE trial compared risankizumab with ustekinumab in a head-to-head design. At week 24, risankizumab was non-inferior for clinical remission (59% vs. 40%) and superior in achieving endoscopic remission.^14^ However, the trial was open-label and did not assess longer-term outcomes, such as disease progression or complications. Although clinical trials remain the gold standard in comparing therapeutic agents, their findings do not always replicate in real-world clinical data.

Therefore, we conducted the first retrospective real-world cohort study using electronic health record (EHR) data to compare risankizumab to ustekinumab. This study assesses long-term effectiveness outcomes, including disease progression, complications, and treatment-related adverse events, in both advanced therapy-exposed and advanced therapy-naïve CD cohorts.

## Methods

### Database

This retrospective cohort study uses data from TriNetX, a global clinical research platform based in Cambridge, Massachusetts. The primary source of clinical data for the network is real-time EHRs from healthcare organizations (HCOs). Clinical features are extracted using an integrated natural language processing (NLP) system, and the data undergo quality checks to ensure accuracy and reliability. TriNetX’s data cleansing process removes records that do not meet its high-quality standards. To protect patient privacy, TriNetX hides patient counts in datasets with fewer than 10 individuals. A trained standards expert validates the de-identification process and complies with the Health Insurance Portability and Accountability Act (HIPAA). These safety measures ensure secure, privacy-compliant data transmission across networks, enabling high-quality research.

### Study population and patient eligibility

We conducted real-time searches and analyses using the TriNetX platform through the Global Collaborative Network from inception to March 29, 2026. This network includes data from approximately 153 266 389 patients across 171 HCOs worldwide. We selected adult patients (≥18 years) diagnosed with CD (K50). We excluded those with comorbid diagnoses of UC, psoriasis, systemic lupus erythematosus (SLE), rheumatoid arthritis, ankylosing spondylitis, multiple sclerosis, or arteritis, given that these conditions may independently influence biologic selection, corticosteroid consumption, hospitalization, surgical risk, and treatment switching irrespective of CD activity. Restricting the cohorts aimed to enhance internal validity by minimizing confounding by indication and avoiding outcome misclassification, thereby improving specificity for CD-driven treatment decisions. Data were analyzed on 2 separate cohorts: (1) Advanced therapy-exposed patients with CD who received risankizumab or ustekinumab and (2) Advanced therapy-naïve patients with CD who received either risankizumab or ustekinumab. The advanced therapy-exposed cohort was defined as receiving at least one of the following medications prior to starting risankizumab or ustekinumab (ie, index date): infliximab, adalimumab, certolizumab pegol, golimumab, vedolizumab, upadacitinib, tofacitinib, risankizumab, or ustekinumab. On the other hand, the advanced therapy-naïve cohort included CD patients with no documented prior exposure to any of the mentioned advanced therapies before the index date; therefore, risankizumab or ustekinumab served as the first advanced therapy in this cohort. The search queries used to identify each treatment group in each cohort are presented in detail in Supplementary Appendices S1.1 and S1.2.

### Outcomes

We assessed both primary and secondary outcomes at 12 and 24 months. The primary outcomes included frequency of corticosteroid use, hospitalization, and emergency department (ED) visits; rate of intestinal surgery; rate of intestinal complications (incidence of obstruction or fistula formation); rate of patients with C-reactive protein (CRP) ≥ 10 mg/L; and the number of patients switching to another advanced therapy. The secondary outcomes included the incidence of major adverse cardiovascular events (MACE), venous thromboembolism (VTE), and opportunistic infections.

Corticosteroid use was defined by the RxNorm code for prednisone, budesonide, prednisolone, methylprednisolone, dexamethasone, hydrocortisone, or betamethasone. The hospitalization rate was defined using the CPT code for Hospital Inpatient and Observation Care Services, or a specific code for the Inpatient Encounter, as listed on the website. ED visits were defined using a specific code for Emergency from the website. Intestinal surgery was defined as any surgery on the small intestine, large intestine, or anal canal identified using CPT codes or ICD-10-PCS codes. Intestinal complications involving both the small and large intestines were defined, and the incidence of obstruction and fistula formation were separately analyzed for each. A switch to another advanced therapy was defined as the use of infliximab, adalimumab, certolizumab pegol, golimumab, vedolizumab, tofacitinib, or upadacitinib after risankizumab or ustekinumab during the 24-month follow-up period. VTE was defined using ICD-10-CM codes for pulmonary embolism or embolism and thrombosis of the deep lower vein. MACE was defined for acute myocardial infarction, cerebral infarction, heart failure, or death. Opportunistic infection was defined using ICD-10-CM codes for herpes zoster, toxoplasmosis, tuberculosis, nocardiosis, enterocolitis with Clostridium difficile, Legionnaires’ disease, listeriosis, pneumocystosis, candidiasis, aspergillosis, or cytomegalovirus. Full details of the codes used to define each outcome are provided in Supplementary Appendix S1.3.

### Statistical analysis

The analyses were conducted using the TriNetX platform, a web-based application that provides real-time analytics. Baseline characteristics are presented as the mean (standard deviation) for continuous variables and as proportions for categorical variables. To achieve a covariate balance between the 2 groups, we used a one-to-one (1:1) propensity score matching (PSM) approach. Covariates were selected a priori based on clinical relevance and their potential association with both treatment assignment and outcomes. These included age at index, gender, ethnicity, race, diabetes mellitus (DM), history of nicotine dependence, overweight, obesity, CD in the small intestine, large intestine, or both, history of intestinal obstruction and fistula due to CD within the small and large intestines, recurrent oral aphthae, erythema nodosum, pyoderma gangrenosum, iridocyclitis, arthralgia, history of surgical procedures on the gastrointestinal system, and prior treatment with corticosteroids, azathioprine, methotrexate, mercaptopurine, mesalamine, sulfasalazine, tacrolimus, infliximab, adalimumab, certolizumab pegol, golimumab, upadacitinib, vedolizumab, risankizumab, ustekinumab, and tofacitinib. Other covariates included hemoglobin, body mass index, leukocytes, albumin, and CRP. Comorbidities such as DM were included in the matching process because they may independently affect hospitalization risk, infection susceptibility, corticosteroid exposure, surgical outcomes, and cardiovascular adverse events. Their inclusion aimed to reduce confounding related to baseline comorbidity burden.^15–17^ Race and ethnicity were incorporated as categorical variables because they may influence healthcare access, treatment allocation, corticosteroid use, and risks of hospitalization, fistula formation, and surgery.^18–20^ Extraintestinal manifestations were included as baseline covariates because they reflect systemic inflammatory burden and a more severe CD phenotype and may influence biologic selection and, independently, affect corticosteroid use, hospitalization, and inflammatory markers. In the absence of validated activity indices (eg, CDAI) or endoscopic scoring within TriNetX, these variables serve as real-world proxies for disease severity, aiming to reduce confounding by indication. Hemoglobin and albumin were included as covariates, as they may reflect disease severity through chronic inflammation, nutritional deficiency, or ongoing gastrointestinal blood loss, which may influence adverse clinical outcomes.^21^^,^^22^

Additionally, CRP and leukocyte count were included to capture complementary aspects of systemic inflammation. Propensity scores for patients were estimated using logistic regression with user-defined covariates. Patients were matched using a greedy nearest-neighbor algorithm with a caliper width of 0.1 pooled standardized mean difference (SMD) to reduce algorithmic bias; the platform also employed random row rearrangement during matching. Covariate balance between matched groups was evaluated using the SMD; values ≤ 0.1 indicated minimal imbalance. In TriNetX, analyses are conducted using only available EHR data, so patients with missing laboratory values did not contribute to the reported variables, and no automatic imputation was performed. Outcomes were evaluated as cumulative incidence at predefined 12- and 24-month follow-up intervals from the index date and compared after matching using odds ratios (ORs) with 95% confidence intervals (CIs). A *P*-value <.05 was considered statistically significant. We used R software (v4.4.1) in conjunction with Python (v3.13.3) for data visualization.

## Results

### Advanced therapy-exposed cohort

**Figure 1:**
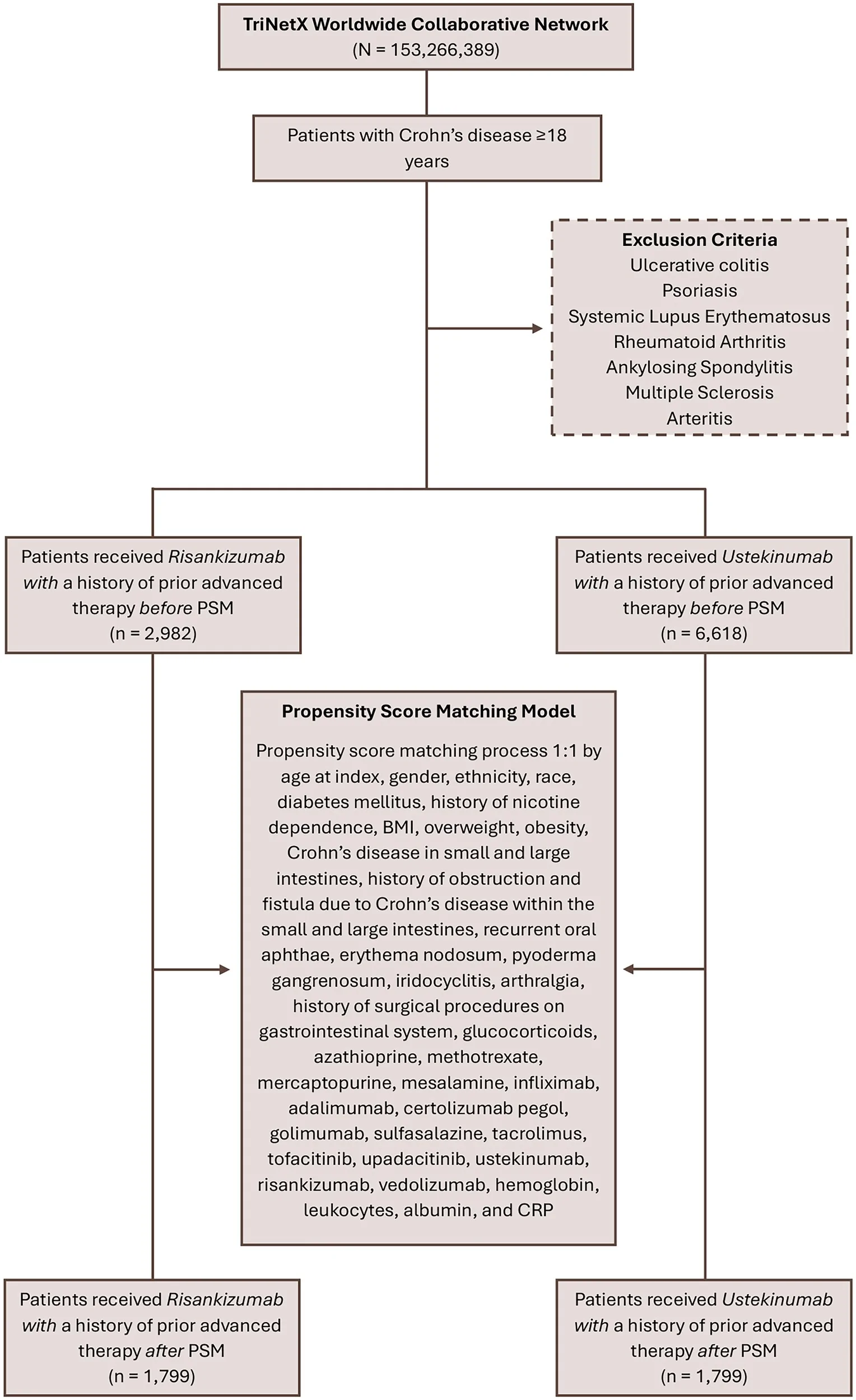
Flow diagram of patients received risankizumab and ustekinumab in advanced therapy–exposed cohort. Abbreviations: BMI: body mass index; CRP: C-reactive protein; PSM: propensity score matching.

We identified 456 941 patients with CD aged 18 or older. Among them, 2982 were in the risankizumab group, and 6618 were in the ustekinumab group. After PSM, each group consisted of 1799 patients. Figure 1 illustrates our selection and matching process. In the risankizumab group, the mean age at index was 42.4 ± 16.6 years, whereas 42.5 ± 17.0 years in the ustekinumab group. The risankizumab group included 951 females (52.9%), while the ustekinumab group comprised 969 (53.9%). Table 1 provides a detailed summary of demographic characteristics, comorbid conditions, laboratory parameters, and medication used before and after PSM in the advanced therapy-exposed cohort.

**Table 1:** Baseline characteristics of patients in the risankizumab and ustekinumab groups of the advanced therapy–exposed cohort.

Covariate	Advanced therapy–exposed cohort					
	Before PSM			After PSM		
	Risankizumab (*N* = 2982)	Ustekinumab (*N* = 6618)	SMD	Risankizumab (*N* = 1799)	Ustekinumab (*N* = 1799)	SMD
**Age at index**	43.4 ± 16.6	40.6 ± 16.8	0.172	42.4 ± 16.6	42.5 ± 17.0	0.010
**Gender**						
**Female**	1640 (55.0)	3659 (55.3)	0.006	951 (52.9)	969 (53.9)	0.020
**Male**	1339 (44.9)	2953 (44.6)	0.006	846 (47.0)	828 (46.0)	0.020
**Ethnicity**						
**Hispanic or Latino**	96 (3.2)	204 (3.1)	0.008	52 (2.9)	49 (2.7)	0.010
**Not Hispanic or Latino**	2248 (75.4)	4267 (64.5)	0.240	1351 (75.1)	1344 (74.7)	0.009
**Unknown**	638 (21.4)	2147 (32.4)	0.251	396 (22.0)	406 (22.6)	0.013
**Race**						
**White**	2367 (79.4)	4812 (72.7)	0.157	1405 (78.1)	1408 (78.3)	0.004
**Black or African American**	242 (8.1)	555 (8.4)	0.010	160 (8.9)	158 (8.8)	0.004
**Asian**	78 (2.6)	135 (2.0)	0.038	51 (2.8)	52 (2.9)	0.003
**American Indian or Alaska Native**	16 (0.5)	20 (0.3)	0.036	10 (0.6)	10 (0.6)	<0.001
**Native Hawaiian or Other Pacific Islander**	10 (0.3)	10 (0.2)	0.037	10 (0.6)	10 (0.6)	<0.001
**Other**	83 (2.8)	189 (2.9)	0.004	46 (2.6)	50 (2.8)	0.014
**Unknown**	191 (6.4)	901 (13.6)	0.242	124 (6.9)	121 (6.7)	0.007
**Diagnosis**						
**Diabetes mellitus**	239 (8.0)	387 (5.8)	0.085	128 (7.1)	126 (7.0)	0.004
**History of nicotine dependence**	561 (18.8)	824 (12.5)	0.176	310 (17.2)	307 (17.1)	0.004
**Crohn’s disease of small intestine**	1962 (65.8)	3822 (57.8)	0.166	1108 (61.6)	1122 (62.4)	0.016
**Crohn’s disease of large intestine**	1723 (57.8)	3390 (51.2)	0.132	999 (55.5)	994 (55.3)	0.006
**Crohn’s disease of both small and large intestine**	2002 (67.1)	3950 (59.7)	0.155	1152 (64.0)	1166 (64.8)	0.016
**Crohn’s disease of both small and large intestine with intestinal obstruction**	606 (20.3)	769 (11.6)	0.239	309 (17.2)	306 (17.0)	0.004
**Crohn’s disease of both small and large intestine with fistula**	523 (17.5)	804 (12.1)	0.152	273 (15.2)	262 (14.6)	0.017
**Recurrent oral aphthae**	70 (2.3)	105 (1.6)	0.055	40 (2.2)	39 (2.2)	0.004
**Erythema nodosum**	42 (1.4)	92 (1.4)	0.002	24 (1.3)	24 (1.3)	<0.001
**Pyoderma gangrenosum**	43 (1.4)	70 (1.1)	0.035	20 (1.1)	20 (1.1)	<0.001
**Iridocyclitis**	26 (0.9)	66 (1.0)	0.013	13 (0.7)	17 (0.9)	0.024
**Arthralgia**	916 (30.7)	1514 (22.9)	0.178	543 (30.2)	536 (29.8)	0.008
**Overweight and obesity**	628 (21.1)	849 (12.8)	0.221	360 (20.0)	330 (18.3)	0.042
**Procedures**						
**Surgery on gastrointestinal system**	1081 (36.3)	2025 (30.6)	0.120	608 (33.8)	578 (32.1)	0.035
**Surgical procedures on the digestive system**	2103 (70.5)	3664 (55.4)	0.318	1213 (67.4)	1218 (67.7)	0.006
**Medications**						
**Azathioprine**	606 (20.3)	1384 (20.9)	0.015	362 (20.1)	354 (19.7)	0.011
**Methotrexate**	435 (14.6)	916 (13.8)	0.021	237 (13.2)	240 (13.3)	0.005
**Mercaptopurine**	336 (11.3)	765 (11.6)	0.009	178 (9.9)	161 (8.9)	0.032
**Mesalamine**	805 (27.0)	1810 (27.3)	0.008	479 (26.6)	471 (26.2)	0.010
**Sulfasalazine**	140 (4.7)	265 (4.0)	0.034	73 (4.1)	83 (4.6)	0.027
**Tacrolimus**	103 (3.5)	166 (2.5)	0.056	50 (2.8)	57 (3.2)	0.023
**Glucocorticoids**	2691 (90.2)	5354 (80.9)	0.268	1580 (87.8)	1583 (88.0)	0.005
**Infliximab**	952 (31.9)	2290 (34.6)	0.057	636 (35.4)	630 (35.0)	0.007
**Adalimumab**	1373 (46.0)	3804 (57.5)	0.230	970 (53.9)	937 (52.1)	0.037
**Certolizumab pegol**	218 (7.3)	600 (9.1)	0.064	123 (6.8)	122 (6.8)	0.002
**Golimumab**	19 (0.6)	26 (0.4)	0.034	10 (0.6)	10 (0.6)	<0.001
**Upadacitinib**	199 (6.7)	43 (0.6)	0.325	56 (3.1)	40 (2.2)	0.055
**Vedolizumab**	612 (20.5)	1177 (17.8)	0.070	349 (19.4)	352 (19.6)	0.004
**Risankizumab**	36 (1.2)	69 (1.0)	0.016	24 (1.3)	34 (1.9)	0.044
**Ustekinumab**	1265 (42.4)	145 (2.2)	1.104	133 (7.4)	144 (8.0)	0.023
**Tofacitinib**	17 (0.6)	17 (0.3)	0.049	10 (0.6)	10 (0.6)	<0.001
**Laboratory parameters**						
**Hemoglobin, g/dL**	12.8 ± 1.9	12.7 ± 2.0	0.066	12.9 ± 1.9	12.8 ± 2.0	0.091
**Albumin, g/dL**	4.0 ± 0.5	3.9 ± 0.6	0.122	4.0 ± 0.5	3.9 ± 0.6	0.125
**BMI, kg/m^2^**	27.4 ± 7.0	26.3 ± 6.8	0.152	27.5 ± 7.0	27.2 ± 7.1	0.052
**Leukocytes, 10^3^/μL**	28.8 ± 306.0	17.6 ± 200.9	0.043	22.5 ± 251.4	18.9 ± 207.4	0.015
**CRP, mg/L**	19.7 ± 36.0	21.0 ± 37.4	0.035	21.1 ± 39.0	20.9 ± 38.4	0.006

Data are presented as mean ± standard deviation for continuous covariates and as counts and percentage for binary covariates.

Abbreviations: BMI: body mass index; CRP: C-reactive protein; PSM: propensity score matching; SMD: standardized mean difference.

**Figure 2:**
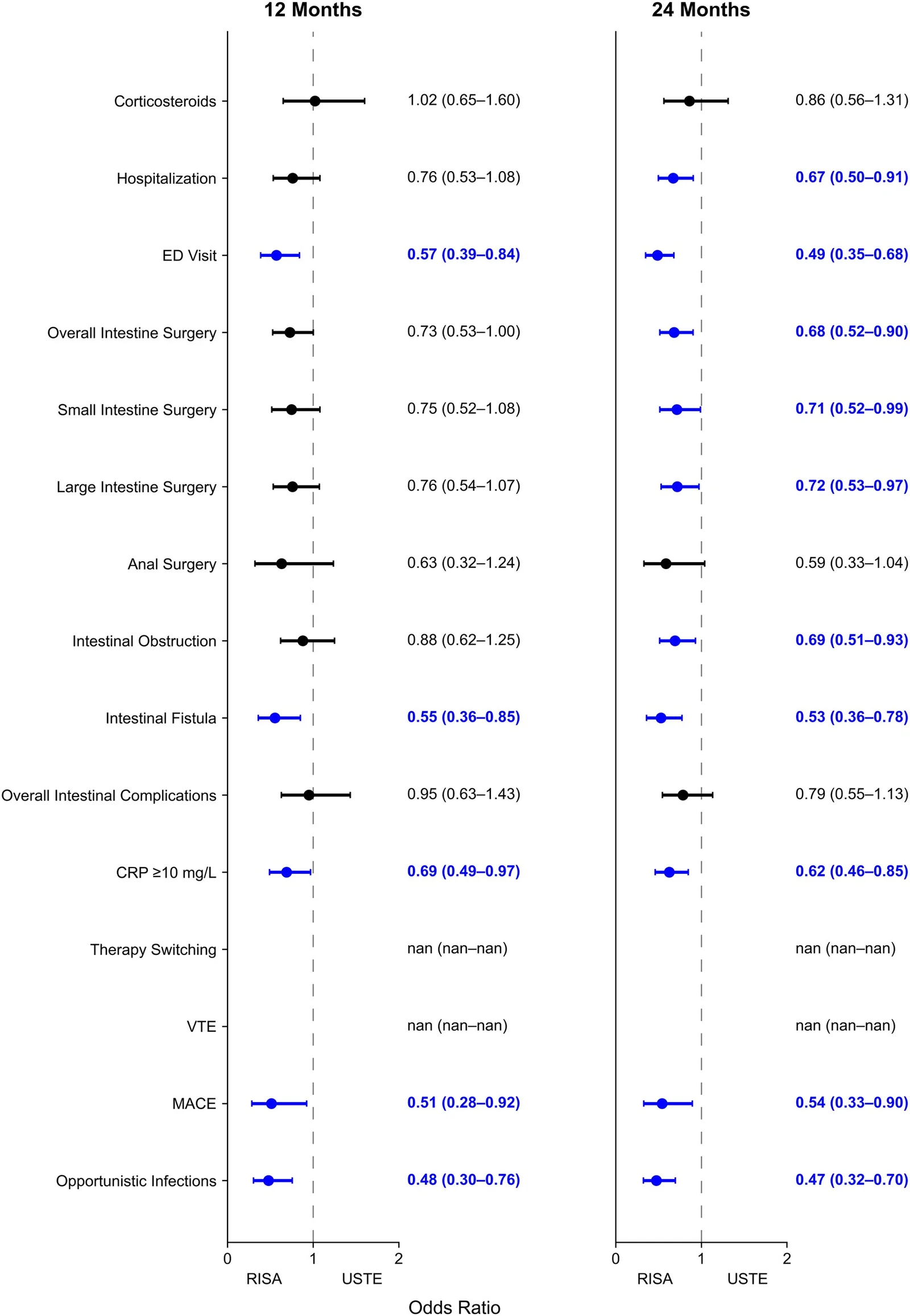
Primary and secondary outcomes at 12 and 24 months between risankizumab and ustekinumab in advanced therapy–exposed cohort. Abbreviations: CRP: C-reactive protein; ED: emergency department; MACE: major adverse cardiovascular events; RISA: risankizumab; USTE: ustekinumab; VTE: venous thromboembolism.

**Figure 3:**
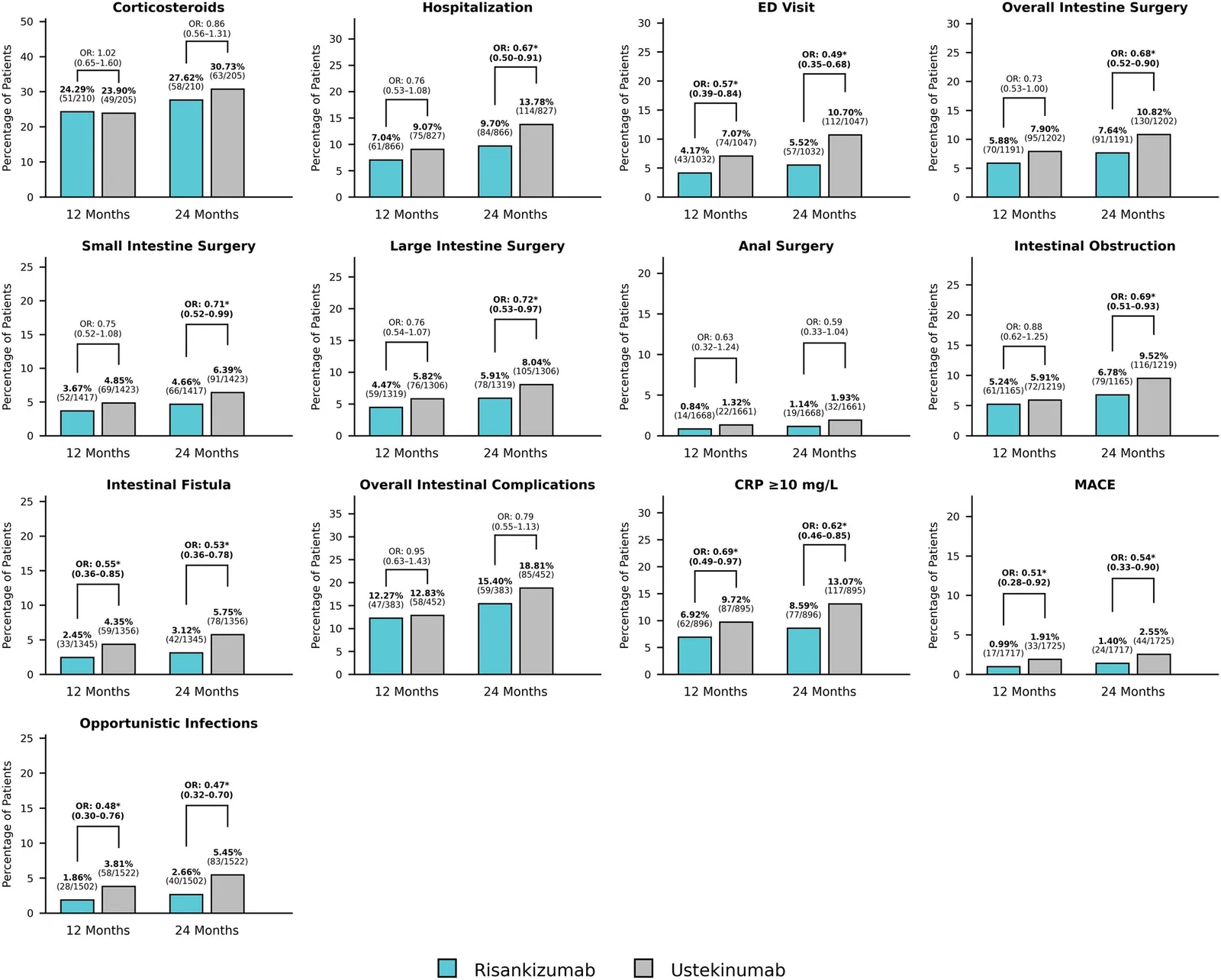
Event rates of primary and secondary outcomes at 12 and 24 months between risankizumab and ustekinumab in the advanced therapy–exposed cohort. Abbreviations: CRP: C-reactive protein; ED: emergency department; MACE: major adverse cardiovascular events; OR: odds ratio. *Statistically significant estimate.

At 12 months, the risankizumab group had a statistically significantly lower rate of ED visits (OR = 0.57, 95% CI, 0.39-0.84) and a lower number of patients who developed intestinal fistula (OR = 0.55, 95% CI, 0.36-0.85) and patients with CRP ≥10 mg/L (OR = 0.69, 95% CI, 0.49-0.97), MACE (OR = 0.51, 95% CI, 0.28-0.92), and opportunistic infections (OR = 0.48, 95% CI, 0.30-076) compared with the ustekinumab group. No statistically significant differences were found between groups in corticosteroid use, hospitalization rate, surgery rate, overall intestinal complications, and intestinal obstruction (Figures 2 and 3). We were unable to assess switching to another advanced therapy and VTE due to the extremely low count of patients, which TriNetX inherently obscures for patient privacy purposes.

At 24 months, compared with ustekinumab group, the risankizumab group demonstrated a statistically significantly lower odds of hospitalization (OR = 0.67, 95% CI, 0.50-0.91), ED visits (OR = 0.49, 95% CI, 0.35-0.68), overall intestinal surgery (OR = 0.68, 95% CI, 0.52-0.90), small intestine surgery (OR = 0.71, 95% CI, 0.52-0.99), large intestine surgery (OR = 0.72, 95% CI, 0.53-0.97), intestinal obstruction (OR = 0.69, 95% CI, 0.51-0.93), intestinal fistula (OR = 0.53, 95% CI, 0.36-0.78), MACE (OR = 0.54, 95% CI, 0.33-0.90), and opportunistic infections (OR = 0.47, 95% CI, 0.32-0.70), with lower proportion of patients with CRP ≥10 mg/L (OR: 0.62, 95% CI, 0.46-0.85). No statistically significant differences were found between groups in corticosteroid use, anal surgery, or overall complications of the intestine (Figures 2 and 3). As for the 12 months, we were unable to assess switching to another advanced therapy and VTE at 24 months.

### Advanced therapy-naïve cohort

**Figure 4:**
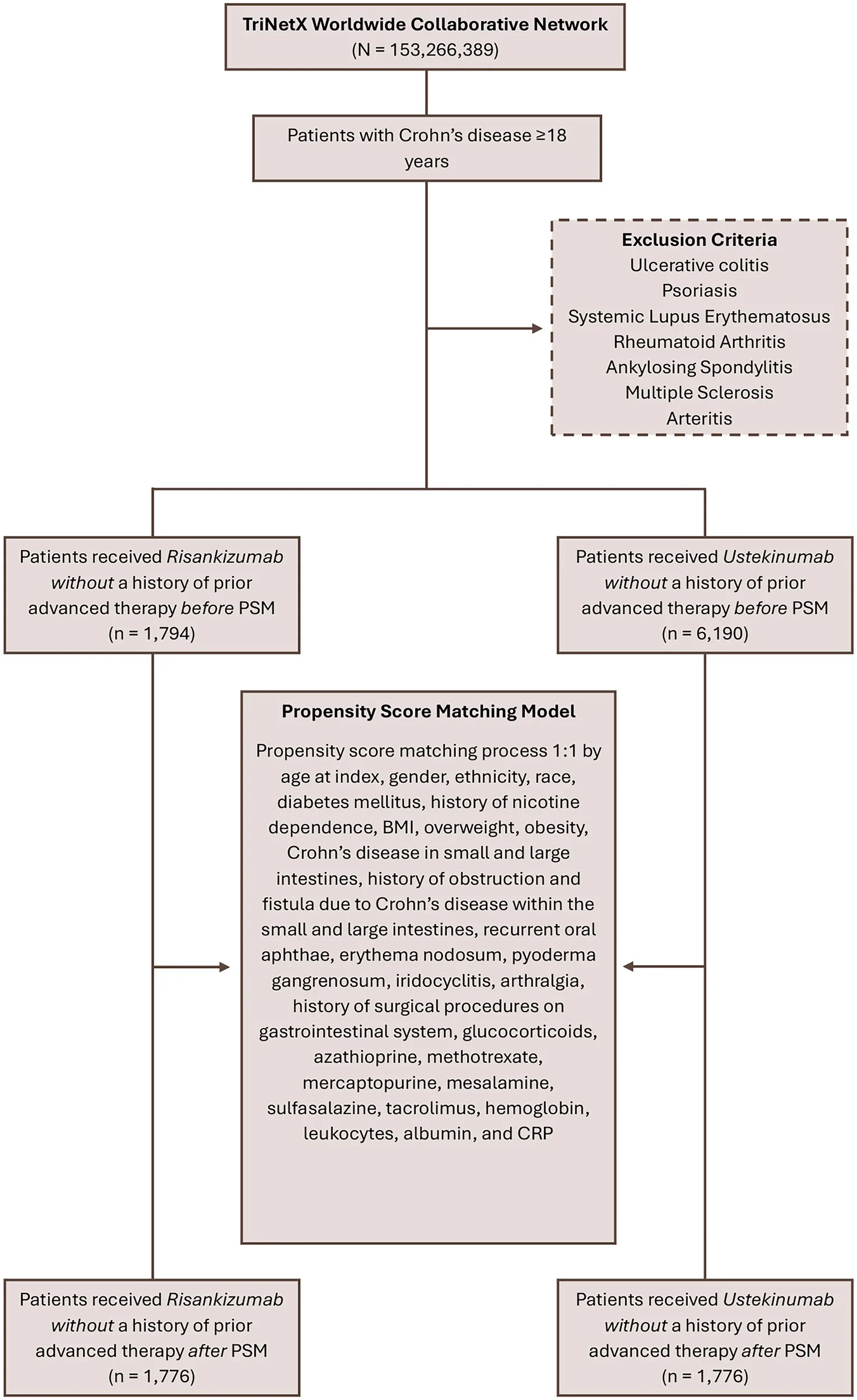
Flow diagram of patients received risankizumab and ustekinumab in advanced therapy–naïve cohort. Abbreviations: BMI: body mass index; CRP: C-reactive protein; PSM: propensity score matching.

We identified 456 941 patients with CD aged 18 or older. Among them, 1794 were in the risankizumab group, and 6190 were in the ustekinumab group. After PSM, each group consisted of 1776 patients. Figure 4 illustrates our selection and matching process. In the risankizumab group, the mean age was 43.8 ± 16.9 years, whereas 43.3 ± 16.7 years in the ustekinumab group. The risankizumab group included 1012 females (57%), while the ustekinumab group comprised 981 females (55.2%). Table 2 provides a detailed summary of demographic characteristics, comorbid conditions, laboratory parameters, and medication use before and after PSM in the advanced therapy-naïve cohort.

**Table 2:** Baseline characteristics of patients in the risankizumab and ustekinumab groups of the advanced therapy–naïve cohort.

Covariate	Advanced therapy–naïve cohort					
	Before PSM			After PSM		
	Risankizumab (*N* = 1794)	Ustekinumab (*N* = 6190)	SMD	Risankizumab (*N* = 1776)	Ustekinumab (*N* = 1776)	SMD
**Age at index**	43.8 ± 16.9	42.6 ± 16.8	0.068	43.8 ± 16.9	43.3 ± 16.7	0.026
**Gender**						
**Female**	1020 (56.9)	3493 (56.4)	0.009	1012 (57.0)	981 (55.2)	0.035
**Male**	774 (43.1)	2695 (43.5)	0.008	764 (43.0)	795 (44.8)	0.035
**Ethnicity**						
**Hispanic or Latino**	78 (4.3)	207 (3.3)	0.052	74 (4.2)	83 (4.7)	0.025
**Not Hispanic or Latino**	1343 (74.9)	4185 (67.6)	0.161	1330 (74.9)	1328 (74.8)	0.003
**Unknown**	373 (20.8)	1798 (29.0)	0.192	372 (20.9)	365 (20.6)	0.010
**Race**						
**White**	1391 (77.5)	4536 (73.3)	0.099	1376 (77.5)	1390 (78.3)	0.019
**Black or African American**	146 (8.1)	433 (7.0)	0.043	144 (8.1)	133 (7.5)	0.023
**Asian**	48 (2.7)	113 (1.8)	0.057	47 (2.6)	43 (2.4)	0.014
**American Indian or Alaska Native**	10 (0.6)	16 (0.3)	0.047	10 (0.6)	10 (0.6)	<0.001
**Native Hawaiian or Other Pacific Islander**	10 (0.6)	10 (0.2)	0.066	10 (0.6)	10 (0.6)	<0.001
**Other**	63 (3.5)	232 (3.7)	0.013	63 (3.5)	58 (3.3)	0.016
**Unknown**	139 (7.7)	853 (13.8)	0.196	139 (7.8)	144 (8.1)	0.010
**Diagnosis**						
**Diabetes mellitus**	113 (6.3)	315 (5.1)	0.052	110 (6.2)	106 (6.0)	0.009
**History of nicotine dependence**	225 (12.5)	491 (7.9)	0.153	215 (12.1)	208 (11.7)	0.012
**Crohn’s disease of small intestine**	844 (47.0)	2401 (38.8)	0.167	832 (46.8)	808 (45.5)	0.027
**Crohn’s disease of large intestine**	666 (37.1)	1895 (30.6)	0.138	660 (37.2)	641 (36.1)	0.022
**Crohn’s disease of both small and large intestine**	750 (41.8)	2280 (36.8)	0.102	739 (41.6)	750 (42.2)	0.013
**Crohn’s disease of both small and large intestine with intestinal obstruction**	164 (9.1)	384 (6.2)	0.111	160 (9.0)	160 (9.0)	<0.001
**Crohn’s disease of both small and large intestine with fistula**	120 (6.7)	396 (6.4)	0.012	120 (6.8)	124 (7.0)	0.009
**Recurrent oral aphthae**	29 (1.6)	36 (0.6)	0.099	21 (1.2)	25 (1.4)	0.020
**Erythema nodosum**	10 (0.6)	44 (0.7)	0.019	10 (0.6)	10 (0.6)	<0.001
**Pyoderma gangrenosum**	13 (0.7)	34 (0.5)	0.022	11 (0.6)	14 (0.8)	0.020
**Iridocyclitis**	12 (0.7)	35 (0.6)	0.013	11 (0.6)	10 (0.6)	0.007
**Arthralgia**	378 (21.1)	946 (15.3)	0.150	369 (20.8)	382 (21.5)	0.018
**Overweight and obesity**	294 (16.4)	599 (9.7)	0.200	282 (15.9)	281 (15.8)	0.002
**Procedures**						
**Surgery on gastrointestinal system**	326 (18.2)	1004 (16.2)	0.052	326 (18.4)	321 (18.1)	0.007
**Surgical procedures on the digestive system**	889 (49.6)	2158 (34.9)	0.301	872 (49.1)	871 (49.0)	0.001
**Medications**						
**Azathioprine**	94 (5.2)	466 (7.5)	0.094	94 (5.3)	89 (5.0)	0.013
**Methotrexate**	37 (2.1)	218 (3.5)	0.089	37 (2.1)	49 (2.8)	0.044
**Mercaptopurine**	76 (4.2)	278 (4.5)	0.012	76 (4.3)	73 (4.1)	0.008
**Mesalamine**	285 (15.9)	936 (15.1)	0.021	282 (15.9)	288 (16.2)	0.009
**Sulfasalazine**	43 (2.4)	133 (2.1)	0.017	43 (2.4)	47 (2.6)	0.014
**Tacrolimus**	25 (1.4)	62 (1.0)	0.036	23 (1.3)	24 (1.4)	0.005
**Glucocorticoids**	1161 (64.7)	3240 (52.3)	0.253	1145 (64.5)	1145 (64.5)	<0.001
**Infliximab**	–	–	–	–	–	–
**Adalimumab**	–	–	–	–	–	–
**Certolizumab pegol**	–	–	–	–	–	–
**Golimumab**	–	–	–	–	–	–
**Upadacitinib**	–	–	–	–	–	–
**Vedolizumab**	–	–	–	–	–	–
**Risankizumab**	–	–	–	–	–	–
**Ustekinumab**	–	–	–	–	–	–
**Tofacitinib**	–	–	–	–	–	–
**Laboratory Parameters**						
**Hemoglobin, g/dL**	12.9 ± 1.9	12.8 ± 2.1	0.064	12.9 ± 1.9	12.8 ± 2.0	0.042
**Albumin, g/dL**	4.0 ± 0.6	4.0 ± 0.6	0.077	4.0 ± 0.6	4.0 ± 0.6	0.085
**BMI, kg/m^2^**	27.3 ± 7.0	26.7 ± 6.6	0.096	27.3 ± 7.0	27.0 ± 6.8	0.034
**Leukocytes, 10^3^/μL**	15.9 ± 192.4	14.4 ± 170.1	0.008	16.0 ± 193.7	11.9 ± 131.3	0.025
**CRP, mg/L**	22.3 ± 42.9	20.4 ± 36.3	0.047	22.3 ± 43.1	20.2 ± 36.1	0.055

Data are presented as mean ± standard deviation for continuous covariates and as counts and percentage for binary covariates.

Abbreviations: BMI: body mass index; CRP: C-reactive protein; PSM: propensity score matching; SMD: standardized mean difference.

**Figure 5:**
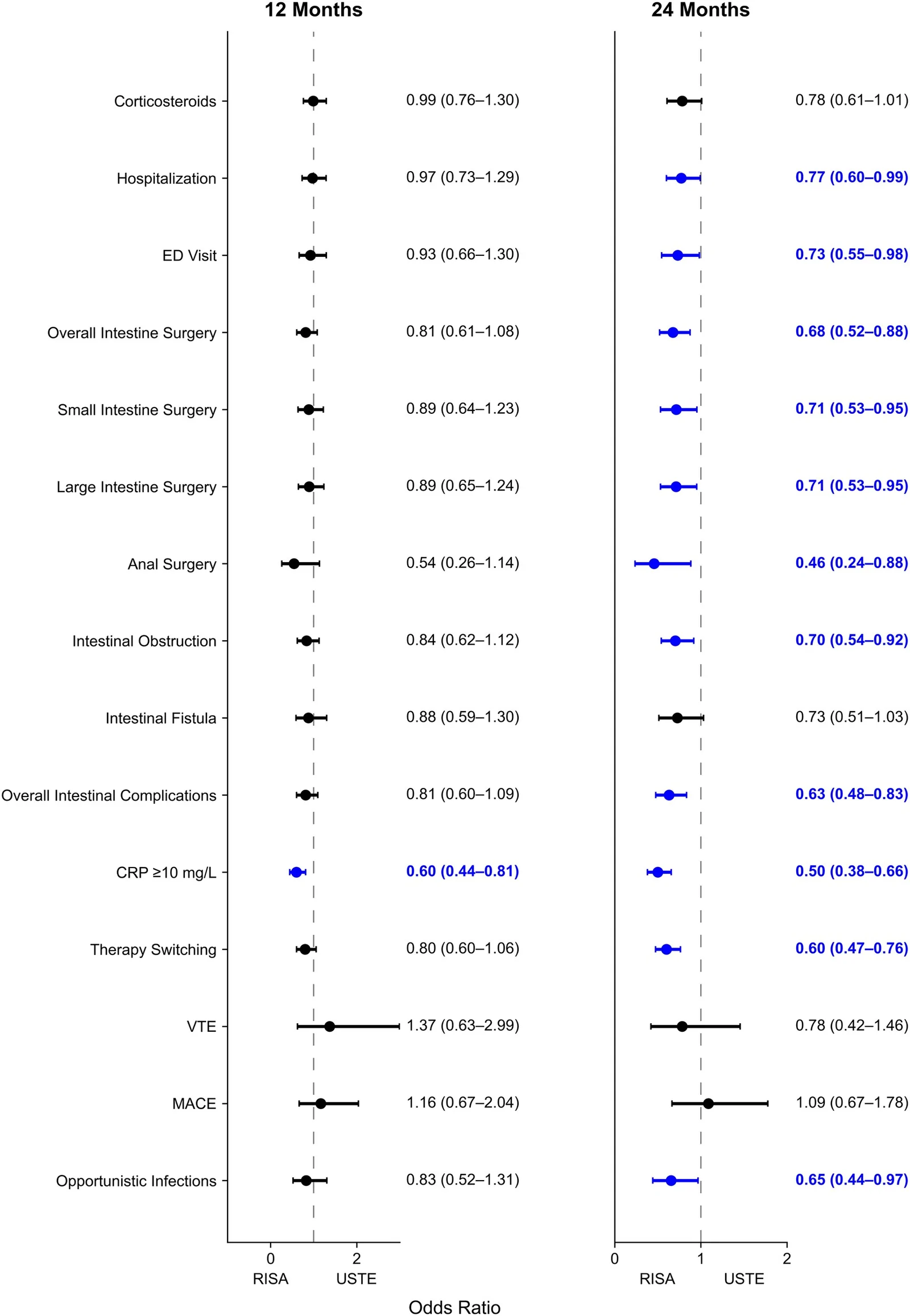
Primary and secondary outcomes at 12 and 24 months between risankizumab and ustekinumab in advanced therapy–naïve cohort. Abbreviations: CRP: C-reactive protein; ED: emergency department; MACE: major adverse cardiovascular events; RISA: risankizumab; USTE: ustekinumab; VTE: venous thromboembolism.

**Figure 6:**
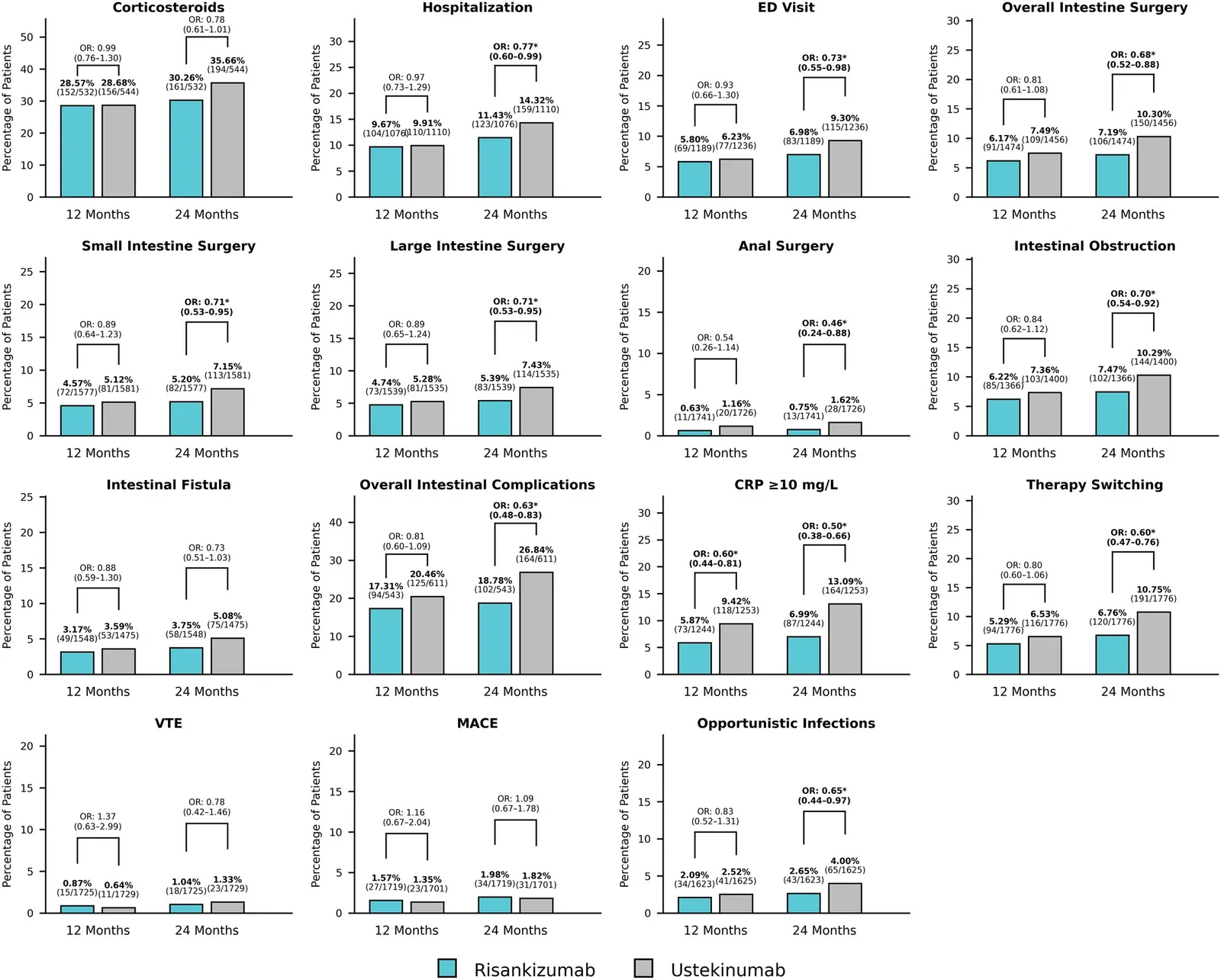
Event rates of primary and secondary outcomes at 12 and 24 months between risankizumab and ustekinumab in the advanced therapy–naïve cohort. Abbreviations: CRP: C-reactive protein; ED: emergency department; MACE: major adverse cardiovascular events; OR: odds ratio; VTE: venous thromboembolism. *Statistically significant estimate.

At 12 months, compared with ustekinumab, the risankizumab group had a statistically significantly lower proportion of patients with CRP ≥10 mg/L (OR = 0.60, 95% CI, 0.44-0.81). However, no statistically significant differences were observed between the 2 groups in corticosteroid use, hospitalization, ED visits, intestine surgery, intestine-specific complications, switching to another advanced therapy, VTE, MACE, or opportunistic infections (Figures 5 and 6).

At 24 months, the risankizumab group was associated with a statistically significantly lower odds of hospitalization (OR = 0.77, 95% CI, 0.60-0.99), ED visits (OR = 0.73, 95% CI, 0.55-0.98), overall intestinal surgery (OR = 0.68, 95% CI, 0.52-0.88), small intestine surgery (OR = 0.71, 95% CI, 0.53-0.95), large intestine surgery (OR = 0.71, 95% CI, 0.53-0.95), anal surgery (OR = 0.46, 95% CI, 0.24-0.88), overall complication of intestines (OR = 0.63, 95% CI, 0.48-0.83), intestinal obstruction (OR = 0.70, 95% CI, 0.54-0.92), switching to another advanced therapy (OR = 0.60, 95% CI, 0.47-0.76), and opportunistic infections (OR = 0.65, 95% CI, 0.44-0.97), with lower proportion of patients with CRP ≥10 mg/L (OR = 0.50, 95% CI, 0.38-0.66) compared with the ustekinumab group. No statistically significant differences were observed between the two groups in corticosteroid use, intestinal fistula, VTE, or MACE (Figures 5 and 6).

## Discussion

In this real-world retrospective cohort study, we primarily evaluated effectiveness outcomes, including corticosteroid use, hospitalization, ED visits, surgical rates, complications, inflammatory biomarkers, treatment switching, and opportunistic infections. Among patients with a history of advanced therapy, risankizumab was associated with statistically significantly lower rates of hospitalization, ED visits, intestinal surgery, intestinal obstruction and fistula, CRP ≥10 mg/L, MACE, and opportunistic infections within 24 months compared with ustekinumab. Notably, no statistically significant differences were observed in corticosteroid use, anal surgery, or overall intestine–specific complications between groups. Similarly, among patients without a history of advanced therapy, risankizumab was associated with lower odds of hospitalization, ED visits, intestinal surgery, intestinal obstruction, overall intestine-specific complications, CRP ≥10 mg/L, switching to another advanced therapy, and opportunistic infections within 24 months. However, no significant differences were observed for corticosteroid use, intestinal fistula, VTE, or MACE between groups at the end of the 24 months.

The SEQUENCE trial was a multicenter, open-label, phase IIIb randomized clinical trial that assessed the efficacy of risankizumab compared to ustekinumab under controlled conditions. The trial demonstrated that the risankizumab group achieved higher rates of glucocorticoid-free clinical remission and glucocorticoid-free endoscopic remission at week 48, compared to the ustekinumab group, with percentage-adjusted differences of 20.1% and 15.9%, respectively. Additionally, risankizumab was non-inferior to ustekinumab at week 24 and superior at week 48 in achieving clinical remission, with percentage points of 18.4% and 19.7%, respectively. Moreover, the trial demonstrated higher rates of endoscopic remission and response at week 48 in the risankizumab group, with 15.6% and 23.3% percentage-point increases, respectively.^14^

It is important to emphasize that these endpoints reflect efficacy outcomes measured within a randomized controlled setting, whereas our study assessed real-world effectiveness outcomes. While our real-world study showed that risankizumab was not associated with lower steroid consumption at 24 months in advance-therapy–exposed and naïve cohorts, these differences may reflect variations in patient selection, disease severity, treatment sequencing, and clinical practice patterns rather than a direct inconsistency between trial efficacy and real-world effectiveness.

A matching-adjusted indirect comparison study was conducted using individual patient-level data from 3 CD trials, which demonstrated a statistically significant higher proportion of patients achieved clinical remission, endoscopic response, and endoscopic remission with risankizumab compared with ustekinumab during the induction phase, with percentage differences of 15%, 26%, and 9%, respectively, suggesting superior efficacy under controlled trial conditions. However, at the end of the maintenance phase, there was no statistically significant difference between risankizumab and ustekinumab in terms of clinical remission.^23^ In other autoimmune diseases, randomized trials have demonstrated the superior efficacy of risankizumab compared with ustekinumab in treating moderate-to-severe chronic plaque psoriasis and psoriatic arthritis.^13^^,^^24^^,^^25^

The temporal pattern observed in our advanced therapy-naïve analyses also warrants further discussion. Several real-world cohorts have shown that risankizumab achieves earlier response rates in ustekinumab-naïve patients when evaluated as a single treatment arm.^26^^,^^27^ However, these studies did not include a ustekinumab comparator and therefore cannot address the timing of risankizumab’s comparative benefit over ustekinumab. By contrast, evidence from direct or indirect comparative studies is more consistent with our findings. The SEQUENCE trial, which enrolled exclusively patients with prior anti-TNF exposure, demonstrated an early and sustained advantage of risankizumab over ustekinumab,^14^ whereas a large propensity score-matched real-world comparison restricted to biologic-naïve patients found no significant differences in composite clinical outcomes at either 1 year of follow-up.^28^ One possible explanation is that, among advanced therapy-naïve patients, both agents achieve high rates of early disease control, thereby reducing the ability to detect meaningful between-treatment differences during the initial follow-up period. With longer follow-up, differences in the durability of response, treatment persistence, or secondary loss of response may become more apparent, resulting in greater separation between treatment groups. Nevertheless, these explanations remain speculative, and studies specifically designed to evaluate the temporal pattern of comparative treatment effects according to prior advanced therapy exposure are currently lacking.

The unexpectedly non-significant difference in corticosteroid use between groups differs from findings reported in the SEQUENCE trial and indirect comparisons. Several factors may explain this observation. Although PSM accounted for multiple clinical and laboratory variables, important markers of disease severity, such as endoscopic activity and validated clinical activity indices, were not available in the database, potentially leading to residual confounding. Therefore, residual confounding cannot be excluded. Consequently, first, confounding by indication may have occurred, whereby clinicians preferentially prescribed risankizumab to patients perceived as having more severe or higher-risk disease. Second, channeling bias may have influenced prescribing patterns toward the newer agent. In particular, the comparable corticosteroid use between the risankizumab and ustekinumab groups may further reflect induction-phase management in patients with greater inflammatory burden, necessitating early bridging corticosteroid therapy. Additionally, clinicians may have allowed a longer therapeutic trial before judging response or declaring treatment failure, opting to use short-term corticosteroids to manage symptoms or mild-to-moderate flares rather than switching to other advanced therapy. Indeed, this may be explained by the paradoxically lower switching rate observed in the risankizumab group in the naïve cohort at 24 months. Therefore, comparable results of corticosteroid use in this context may reflect treatment persistence and physician management strategies rather than equivalent effectiveness. Eventually, these findings should be interpreted cautiously.

Regarding safety outcomes, the SEQUENCE trial demonstrated no statistically significant difference between risankizumab and ustekinumab in rates of opportunistic infections, any adverse event, a severe adverse event, or an adverse event leading to treatment discontinuation. However, serious adverse events occurred less frequently in the risankizumab group (10.3% vs. 17.4%), with a percentage-adjusted difference of −7.1%,^14^ reflecting differences observed under controlled trial settings. In our real-world study, we observed no statistically significant differences in overall complications in the exposed cohort. However, we observed statistically significant associations with lower odds of intestinal obstruction, fistula, MACE, and opportunistic infection with risankizumab over 24 months. On the other hand, we noticed no significant differences between groups in intestinal fistula, VTE, or MACE. However, intestinal obstruction, overall complications, and opportunistic infections were significantly lower in the risankizumab group over the 24 months.

Although several outcomes reached statistical significance, the magnitude of the observed associations was generally modest. Therefore, these findings should be interpreted as evidence of modest relative differences between treatments rather than large clinical effects. Given the retrospective observational design and the possibility of residual confounding despite comprehensive PSM, the clinical significance of these differences should be interpreted cautiously.

Our findings provide additional real-world comparative evidence regarding risankizumab and ustekinumab. However, the observed effect sizes were generally modest and should be interpreted in the context of the observational study design and the potential for residual confounding. In patients who have previously tried other advanced therapies, risankizumab may represent a reasonable option for earlier use in the treatment sequence in routine care, as it may reduce hospitalizations and ED visits compared with ustekinumab. Conversely, patients with no history of prior advanced therapies may require careful patient selection and monitoring, as real-world management patterns, including induction bridging therapy and physician preferences, may influence outcomes in this cohort. Additionally, both cohorts of patients may potentially benefit from earlier risankizumab treatment in the treatment sequence with respect to complications and surgery risk over time. Ustekinumab remains a reasonable alternative in advanced therapy-naïve patients within the first 12 months. Furthermore, clinicians should interpret these results as reflecting effectiveness in routine practice and incorporate patient characteristics, disease severity, and treatment history when choosing between risankizumab and ustekinumab. Whether these modest relative differences translate into clinically meaningful long-term benefits remains uncertain and requires confirmation in prospective comparative studies. Longer-term follow-up from the SEQUENCE trial, including anticipated 3- and 5-year analyses, together with prospective real-world comparative studies, will be important to determine the durability and clinical relevance of the observed differences.

### Strengths and limitations

We aimed to highlight differences between risankizumab and ustekinumab across several outcomes over 24 months, representing the first results from large real-world datasets directly comparing the 2 agents, not only in patients with a history of advanced therapies but also in those without such a history. Additionally, we used several covariates to estimate a comprehensive PSM model between the 2 groups. However, the study has several limitations. First, most of the patients were White and non-Hispanic or Latino, which may lead to the results not being representative of the general population. Second, despite our adjustments to the analyses, residual confounding may still exist. The study’s use of electronic clinical records may inadequately represent specific populations, particularly those with limited access to healthcare, potentially leading to biased results. Several limitations inherent to the TriNetX database warrant consideration. TriNetX lacks a standardized variable for disease onset and disease duration. Formal Montreal classification variables are not systematically available as structured data within TriNetX and are typically documented in narrative notes, which precludes standardized phenotypic matching. To address this issue, proxy variables were incorporated into the PSM model, approximating disease location in the small and large intestines and capturing disease behavior through complications such as obstruction, fistula, and prior intestinal surgery, thereby allowing partial phenotypic adjustment.

Additionally, perianal disease could not be reliably adjusted for due to heterogeneous and potentially incomplete ICD coding across institutions. As a result, perianal disease was excluded from the PSM model to avoid potential misclassification bias. However, residual confounding related to unmeasured disease phenotype cannot be entirely excluded. Although we matched groups based on their prior exposure to advanced therapies, we could not account for the number of agents they had received. This is because, while TriNetX offers de-identified patient-level treatment data, the standard analytics output is aggregated, which hindered us from reliably determining the cumulative number of prior agents for each patient. As a result, there may still be residual confounding related to the burden of prior treatments. Additionally, we were unable to address the reasons for switching therapies, as TriNetX does not record clinical reasons for treatment switching, such as primary non-response, loss of response, intolerance, or non-clinical factors. Accordingly, treatment switching was evaluated as a pragmatic real-world composite endpoint reflecting overall treatment non-persistence rather than a definitive measure of therapeutic failure.

Furthermore, TriNetX does not reliably provide key CD endpoints such as clinical remission, steroid-free clinical remission, and endoscopic response/remission. Therefore, our effectiveness assessment relied on proxy outcomes from real-world settings, including corticosteroid use, healthcare utilization, surgery, treatment switching, and biochemical markers. While these measures provide meaningful insights into disease activity and healthcare utilization in the real world, they do not fully substitute for standardized clinical or endoscopic remission endpoints. Lastly, we excluded patients with other immune-mediated inflammatory diseases (eg, psoriasis, rheumatoid arthritis, ankylosing spondylitis) to reduce confounding by indication. While this approach enhances the internal validity of our study, it may limit the generalizability of our findings to patients with CD who have these overlapping comorbidities.

## Conclusions

In this real-world retrospective analysis, risankizumab was associated with modestly more favorable outcomes than ustekinumab, consistent with findings from the SEQUENCE trial, particularly among patients with prior advanced-therapy exposure, where benefits were observed as early as 12 months. In advanced therapy-naïve patients, outcomes were generally comparable at 12 months, with more favorable outcomes observed at 24 months. No major differences in safety outcomes were identified. These findings suggest that risankizumab may represent an effective treatment option, while ustekinumab remains a reasonable alternative, particularly in advanced therapy-naïve patients. Given the absence of data on the clinical remission and endoscopic response or remission, our findings should be interpreted as real-world effectiveness outcomes, and further prospective comparative studies with extended follow-up, including future long-term results from the SEQUENCE trial.

## Supplementary Material

otag096_Supplementary_Data

## Data Availability

The data used in this study were obtained from the TriNetX research network, a global federated health research database that contains de-identified patient-level electronic health record data. Due to data use agreements and privacy regulations, the raw data cannot be publicly shared. Access to the TriNetX platform is limited to licensed users affiliated with participating institutions. Researchers interested in accessing similar data may contact TriNetX (https://trinetx.com/data-sets-analytics/) for more information regarding access and licensing.
