# Supplementary material for: Comparative effectiveness and safety of risankizumab vs ustekinumab in patients with Crohn’s disease: a propensity score–matched multicenter retrospective cohort study
Source: Crohns Colitis 360. 2026 Aug 18;8(3):otag096. doi: 10.1093/crocol/otag096 (PMC13568817; doi:10.1093/crocol/otag096)
Supplement: Supplementary file 1 [file otag096_supplementary_data.docx]

**Supplementary
Materials *of*
Comparative Effectiveness and Safety of Risankizumab Versus Ustekinumab in Patients with Crohn's Disease: A Propensity Score Matched Multicenter Retrospective Cohort Study**

Table of Contents

[Appendix S1. Search Query	3](#_Toc226073226)

[S1.1 Advanced Therapy–Exposed Cohort	3](#_Toc226073227)

[S1.1.1 Query Criteria for Risankizumab Group	3](#_Toc226073228)

[S1.1.2 Query Criteria for Ustekinumab Group	4](#_Toc226073229)

[S1.2 Advanced Therapy–Naïve Cohort	5](#_Toc226073230)

[S1.2.1 Query Criteria for Risankizumab Group	5](#_Toc226073231)

[S1.2.2 Query Criteria for Ustekinumab Group	6](#_Toc226073232)

[S1.3 Query Criteria for Primary and Secondary Outcomes	7](#_Toc226073233)

[Corticosteroids	7](#_Toc226073234)

[Hospitalization	7](#_Toc226073235)

[Emergency Department Visit	7](#_Toc226073236)

[Overall Surgery	7](#_Toc226073237)

[Large Intestine Surgery	9](#_Toc226073238)

[Small Intestine Surgery	10](#_Toc226073239)

[Anal Surgery	10](#_Toc226073240)

[Intestinal Obstruction	11](#_Toc226073241)

[Intestinal Fistula	11](#_Toc226073242)

[Overall Complications of Intestine	11](#_Toc226073243)

[CRP ≥10 mg/L	11](#_Toc226073244)

[Switch to Other Advanced Therapy	11](#_Toc226073245)

[Venous Thromboembolism	11](#_Toc226073246)

[Major Adverse Cardiovascular Events (MACE)	12](#_Toc226073247)

[Opportunistic Infections	12](#_Toc226073248)

Appendix S1. Search Query

S1.1 Advanced Therapy–Exposed Cohort

S1.1.1 Query Criteria for Risankizumab Group

**Patients *must* have:**

Age (Age) (at least 18 years (most recent occurrence)).

**AND**

**Patients *cannot* have:**

***any* of the following:**

Ulcerative colitis (UMLS:ICD10CM:K51); or

Multiple sclerosis (UMLS:ICD10CM:G35); or

Arteritis, unspecified (UMLS:ICD10CM:I77.6); or

Systemic lupus erythematosus (SLE) (UMLS:ICD10CM:M32); or

Psoriasis (UMLS:ICD10CM:L40); or

Ankylosing spondylitis (UMLS:ICD10CM:M45); or

Rheumatoid arthritis with rheumatoid factor (UMLS:ICD10CM:M05); or

Other rheumatoid arthritis (UMLS:ICD10CM:M06).

**AND**

**All the following *must* be satisfied:**

**Crohn's Disease: The terms in this group occurred at any time**

**Patients *must* have:**

Crohn's disease [regional enteritis] (UMLS:ICD10CM:K50).

**Risankizumab: *Any* instance of Risankizumab occurred on or after any instance of Crohn's Disease**

**Patients *must* have:**

***any* of the following:**

risankizumab (NLM:RXNORM:2166040); or

Injection, risankizumab-rzaa, intravenous, 1 mg (UMLS:HCPCS:J2327).

**AND**

**Risankizumab: The terms in this group occurred at any time**

**Patients must have:**

**any of the following:**

risankizumab (NLM:RXNORM:2166040); or

Injection, risankizumab-rzaa, intravenous, 1 mg (UMLS:HCPCS:J2327).

**Advanced Therapy: *Any* instance of Advanced Therapy occurred on or before the first instance of Risankizumab**

**Patients *must* have:**

***any* of the following:**

ustekinumab (NLM:RXNORM:847083); or

Ustekinumab, for intravenous injection, 1 mg (UMLS:HCPCS:J3358); or

Ustekinumab, for subcutaneous injection, 1 mg (UMLS:HCPCS:J3357); or

adalimumab (NLM:RXNORM:327361); or

Ustekinumab, for intravenous injection, 1 mg (deprecated 2017) (UMLS:HCPCS:Q9989); or

Ustekinumab therapy (UMLS:SNOMED:726598003); or

Ustekinumab, for intravenous injection, 1 mg (deprecated 2017) (UMLS:HCPCS:C9487); or

Infliximab therapy (UMLS:SNOMED:713517003); or

infliximab (NLM:RXNORM:191831); or

Infliximab (UMLS:CPT:80230); or

Injection, infliximab, excludes biosimilar, 10 mg (UMLS:HCPCS:J1745); or

Adalimumab (UMLS:CPT:80145); or

Injection, adalimumab, 20 mg (deprecated 2026) (UMLS:HCPCS:J0135); or

Adalimumab therapy (UMLS:SNOMED:722334000); or

certolizumab pegol (NLM:RXNORM:709271); or

Injection, adalimumab, 1 mg (UMLS:HCPCS:J0139); or

golimumab (NLM:RXNORM:819300); or

Injection, golimumab, 1 mg, for intravenous use (UMLS:HCPCS:J1602); or

vedolizumab (NLM:RXNORM:1538097); or

upadacitinib (NLM:RXNORM:2196092); or

Vedolizumab therapy (UMLS:SNOMED:782712008); or

Vedolizumab (UMLS:CPT:80280); or

Injection, vedolizumab, 1 mg (deprecated 2015) (UMLS:HCPCS:C9026); or

tofacitinib (NLM:RXNORM:1357536); or

Injection, vedolizumab, intravenous, 1 mg (UMLS:HCPCS:J3380).

S1.1.2 Query Criteria for Ustekinumab Group

**Patients *must* have:**

Age (Age) (at least 18 years (most recent occurrence)).

**AND**

**Patients *cannot* have:**

***any* of the following:**

Ulcerative colitis (UMLS:ICD10CM:K51); or

Multiple sclerosis (UMLS:ICD10CM:G35); or

Arteritis, unspecified (UMLS:ICD10CM:I77.6); or

Systemic lupus erythematosus (SLE) (UMLS:ICD10CM:M32); or

Psoriasis (UMLS:ICD10CM:L40); or

Ankylosing spondylitis (UMLS:ICD10CM:M45); or

Rheumatoid arthritis with rheumatoid factor (UMLS:ICD10CM:M05); or

Other rheumatoid arthritis (UMLS:ICD10CM:M06).

**AND**

**All the following *must* be satisfied:**

**Crohn's Disease: The terms in this group occurred at any time**

**Patients *must* have:**

Crohn's disease [regional enteritis] (UMLS:ICD10CM:K50).

**Ustekinumab: *Any* instance of Ustekinumab occurred on or after any instance of Crohn's Disease**

**Patients *must* have:**

***any* of the following:**

ustekinumab (NLM:RXNORM:847083); or

Ustekinumab, for intravenous injection, 1 mg (UMLS:HCPCS:J3358); or

Ustekinumab, for subcutaneous injection, 1 mg (UMLS:HCPCS:J3357); or

Ustekinumab, for intravenous injection, 1 mg (deprecated 2017) (UMLS:HCPCS:Q9989); or

Ustekinumab, for intravenous injection, 1 mg (deprecated 2017) (UMLS:HCPCS:C9487); or

Ustekinumab therapy (UMLS:SNOMED:726598003).

**AND**

**Ustekinumab: The terms in this group occurred at any time**

**Patients *must* have:**

***any* of the following:**

ustekinumab (NLM:RXNORM:847083); or

Ustekinumab, for intravenous injection, 1 mg (UMLS:HCPCS:J3358); or

Ustekinumab, for subcutaneous injection, 1 mg (UMLS:HCPCS:J3357); or

Ustekinumab, for intravenous injection, 1 mg (deprecated 2017) (UMLS:HCPCS:Q9989); or

Ustekinumab, for intravenous injection, 1 mg (deprecated 2017) (UMLS:HCPCS:C9487); or

Ustekinumab therapy (UMLS:SNOMED:726598003).

**Advanced Therapy: *Any* instance of Advanced Therapy occurred on or before the first instance of Ustekinumab**

**Patients *must* have:**

***any* of the following:**

risankizumab (NLM:RXNORM:2166040); or

infliximab (NLM:RXNORM:191831); or

Injection, adalimumab, 20 mg (deprecated 2026) (UMLS:HCPCS:J0135); or

Injection, risankizumab-rzaa, intravenous, 1 mg (UMLS:HCPCS:J2327); or

Infliximab (UMLS:CPT:80230); or

Infliximab therapy (UMLS:SNOMED:713517003); or

Injection, infliximab, excludes biosimilar, 10 mg (UMLS:HCPCS:J1745); or

adalimumab (NLM:RXNORM:327361); or

Adalimumab (UMLS:CPT:80145); or

Adalimumab therapy (UMLS:SNOMED:722334000); or

certolizumab pegol (NLM:RXNORM:709271); or

Injection, adalimumab, 1 mg (UMLS:HCPCS:J0139); or

golimumab (NLM:RXNORM:819300); or

Injection, golimumab, 1 mg, for intravenous use (UMLS:HCPCS:J1602); or

vedolizumab (NLM:RXNORM:1538097); or

upadacitinib (NLM:RXNORM:2196092); or

Vedolizumab therapy (UMLS:SNOMED:782712008); or

Vedolizumab (UMLS:CPT:80280); or

Injection, vedolizumab, intravenous, 1 mg (UMLS:HCPCS:J3380); or

Injection, vedolizumab, 1 mg (deprecated 2015) (UMLS:HCPCS:C9026); or

tofacitinib (NLM:RXNORM:1357536).

S1.2 Advanced Therapy–Naïve Cohort

S1.2.1 Query Criteria for Risankizumab Group

**Patients *must* have:**

Age (Age) (at least 18 years (most recent occurrence)).

**AND**

**Patients *cannot* have:**

***any* of the following:**

Ulcerative colitis (UMLS:ICD10CM:K51); or

Multiple sclerosis (UMLS:ICD10CM:G35); or

Arteritis, unspecified (UMLS:ICD10CM:I77.6); or

Systemic lupus erythematosus (SLE) (UMLS:ICD10CM:M32); or

Psoriasis (UMLS:ICD10CM:L40); or

Ankylosing spondylitis (UMLS:ICD10CM:M45); or

Rheumatoid arthritis with rheumatoid factor (UMLS:ICD10CM:M05); or

Other rheumatoid arthritis (UMLS:ICD10CM:M06).

**AND**

**All the following *must* be satisfied:**

**Crohn's Disease: The terms in this group occurred at *any* time**

**Patients *must* have:**

Crohn's disease [regional enteritis] (UMLS:ICD10CM:K50).

**Risankizumab: The *first* instance of Risankizumab occurred on or after any instance of Crohn's Disease**

**Patients *must* have:**

***any* of the following:**

risankizumab (NLM:RXNORM:2166040); or

Injection, risankizumab-rzaa, intravenous, 1 mg (UMLS:HCPCS:J2327).

**AND**

**Risankizumab: The terms in this group occurred at any time**

**Patients *must* have:**

***any* of the following:**

risankizumab (NLM:RXNORM:2166040); or

Injection, risankizumab-rzaa, intravenous, 1 mg (UMLS:HCPCS:J2327).

**Advanced Therapy: *Any* instance of Advanced Therapy occurred on or before the first instance of Risankizumab**

**Patients *cannot* have:**

***any* of the following:**

ustekinumab (NLM:RXNORM:847083); or

Ustekinumab, for intravenous injection, 1 mg (UMLS:HCPCS:J3358); or

Ustekinumab, for subcutaneous injection, 1 mg (UMLS:HCPCS:J3357); or

Ustekinumab, for intravenous injection, 1 mg (deprecated 2017) (UMLS:HCPCS:Q9989); or

Ustekinumab, for intravenous injection, 1 mg (deprecated 2017) (UMLS:HCPCS:C9487); or

Ustekinumab therapy (UMLS:SNOMED:726598003); or

infliximab (NLM:RXNORM:191831); or

Infliximab (UMLS:CPT:80230); or

Infliximab therapy (UMLS:SNOMED:713517003); or

Injection, infliximab, excludes biosimilar, 10 mg (UMLS:HCPCS:J1745); or

Adalimumab (UMLS:CPT:80145); or

adalimumab (NLM:RXNORM:327361); or

Injection, adalimumab, 20 mg (deprecated 2026) (UMLS:HCPCS:J0135); or

Adalimumab therapy (UMLS:SNOMED:722334000); or

certolizumab pegol (NLM:RXNORM:709271); or

Injection, adalimumab, 1 mg (UMLS:HCPCS:J0139); or

golimumab (NLM:RXNORM:819300); or

Injection, golimumab, 1 mg, for intravenous use (UMLS:HCPCS:J1602); or

vedolizumab (NLM:RXNORM:1538097); or

upadacitinib (NLM:RXNORM:2196092); or

Vedolizumab therapy (UMLS:SNOMED:782712008); or

Vedolizumab (UMLS:CPT:80280); or

Injection, vedolizumab, intravenous, 1 mg (UMLS:HCPCS:J3380); or

Injection, vedolizumab, 1 mg (deprecated 2015) (UMLS:HCPCS:C9026); or

tofacitinib (NLM:RXNORM:1357536).

S1.2.2 Query Criteria for Ustekinumab Group

**Patients *must* have:**

Age (Age) (at least 18 years (most recent occurrence)).

**AND**

**Patients *cannot* have:**

***any* of the following:**

Ulcerative colitis (UMLS:ICD10CM:K51); or

Multiple sclerosis (UMLS:ICD10CM:G35); or

Arteritis, unspecified (UMLS:ICD10CM:I77.6); or

Systemic lupus erythematosus (SLE) (UMLS:ICD10CM:M32); or

Psoriasis (UMLS:ICD10CM:L40); or

Ankylosing spondylitis (UMLS:ICD10CM:M45); or

Rheumatoid arthritis with rheumatoid factor (UMLS:ICD10CM:M05); or

Other rheumatoid arthritis (UMLS:ICD10CM:M06).

**AND**

**All the following *must* be satisfied:**

**Crohn's Disease: The terms in this group occurred at any time**

**Patients *must* have:**

Crohn's disease [regional enteritis] (UMLS:ICD10CM:K50).

**Ustekinumab: The *first* instance of Ustekinumab occurred on or after any instance of Crohn's Disease**

**Patients *must* have:**

***any* of the following:**

ustekinumab (NLM:RXNORM:847083); or

Ustekinumab, for intravenous injection, 1 mg (UMLS:HCPCS:J3358); or

Ustekinumab, for subcutaneous injection, 1 mg (UMLS:HCPCS:J3357); or

Ustekinumab, for intravenous injection, 1 mg (deprecated 2017) (UMLS:HCPCS:Q9989); or

Ustekinumab, for intravenous injection, 1 mg (deprecated 2017) (UMLS:HCPCS:C9487); or

Ustekinumab therapy (UMLS:SNOMED:726598003).

**AND**

**Ustekinumab: The terms in this group occurred at any time**

**Patients *must* have:**

***any* of the following:**

ustekinumab (NLM:RXNORM:847083); or

Ustekinumab, for intravenous injection, 1 mg (UMLS:HCPCS:J3358); or

Ustekinumab, for subcutaneous injection, 1 mg (UMLS:HCPCS:J3357); or

Ustekinumab, for intravenous injection, 1 mg (deprecated 2017) (UMLS:HCPCS:Q9989); or

Ustekinumab, for intravenous injection, 1 mg (deprecated 2017) (UMLS:HCPCS:C9487); or

Ustekinumab therapy (UMLS:SNOMED:726598003).

**Advanced Therapy: Any instance of Advanced Therapy occurred on or before the first instance of Ustekinumab**

**Patients *cannot* have:**

***any* of the following:**

risankizumab (NLM:RXNORM:2166040); or

Injection, risankizumab-rzaa, intravenous, 1 mg (UMLS:HCPCS:J2327); or

infliximab (NLM:RXNORM:191831); or

Infliximab (UMLS:CPT:80230); or

Infliximab therapy (UMLS:SNOMED:713517003); or

Injection, infliximab, excludes biosimilar, 10 mg (UMLS:HCPCS:J1745); or

Adalimumab (UMLS:CPT:80145); or

adalimumab (NLM:RXNORM:327361); or

Injection, adalimumab, 20 mg (deprecated 2026) (UMLS:HCPCS:J0135); or

Adalimumab therapy (UMLS:SNOMED:722334000); or

certolizumab pegol (NLM:RXNORM:709271); or

Injection, adalimumab, 1 mg (UMLS:HCPCS:J0139); or

golimumab (NLM:RXNORM:819300); or

Injection, golimumab, 1 mg, for intravenous use (UMLS:HCPCS:J1602); or

vedolizumab (NLM:RXNORM:1538097); or

upadacitinib (NLM:RXNORM:2196092); or

Vedolizumab therapy (UMLS:SNOMED:782712008); or

Vedolizumab (UMLS:CPT:80280); or

Injection, vedolizumab, intravenous, 1 mg (UMLS:HCPCS:J3380); or

Injection, vedolizumab, 1 mg (deprecated 2015) (UMLS:HCPCS:C9026); or

tofacitinib (NLM:RXNORM:1357536).

S1.3 Query Criteria for Primary and Secondary Outcomes

Corticosteroids

**Patients *must* have:**

***any* of the following:**

prednisone (NLM:RXNORM:8640); or

budesonide (NLM:RXNORM:19831); or

prednisolone (NLM:RXNORM:8638); or

methylprednisolone (NLM:RXNORM:6902); or

dexamethasone (NLM:RXNORM:3264); or

hydrocortisone (NLM:RXNORM:5492); or

betamethasone (NLM:RXNORM:1514).

Hospitalization

**Patients *must* have:**

***any* of the following:**

Visit: Inpatient Encounter (UMLS:HL7V3.0:VisitType:IMP); or

Hospital Inpatient and Observation Care Services (UMLS:CPT:1013659).

Emergency Department Visit

**Patients *must* have:**

Visit: Emergency (UMLS:HL7V3.0:VisitType:EMER).

Overall Surgery

**Patients *must* have:**

***any* of the following:**

Laparoscopy, surgical; colectomy, partial, with removal of terminal ileum with ileocolostomy (UMLS:CPT:44205); or

Laparoscopy, surgical; colectomy, total, abdominal, without proctectomy, with ileostomy or ileoproctostomy

(UMLS:CPT:44210); or

Laparoscopy, surgical; colectomy, partial, with end colostomy and closure of distal segment (Hartmann type

procedure) (UMLS:CPT:44206); or

Laparoscopy, surgical; colectomy, partial, with anastomosis, with coloproctostomy (low pelvic anastomosis)

(UMLS:CPT:44207); or

Laparoscopy, surgical; colectomy, total, abdominal, with proctectomy, with ileoanal anastomosis, creation of ileal

reservoir (S or J), with loop ileostomy, includes rectal mucosectomy, when performed (UMLS:CPT:44211); or

Colectomy, partial, with removal of terminal ileum with ileocolostomy (UMLS:CPT:44160); or

Colectomy, partial (UMLS:CPT:1007455); or

Colectomy, total, abdominal, without proctectomy (UMLS:CPT:1007463); or

Gastrointestinal System / Excision / Large Intestine, Left (UMLS:ICD10PCS:0DBG); or

Gastrointestinal System / Excision / Transverse Colon (UMLS:ICD10PCS:0DBL); or

Gastrointestinal System / Excision / Large Intestine, Right (UMLS:ICD10PCS:0DBF); or

Gastrointestinal System / Excision / Ascending Colon (UMLS:ICD10PCS:0DBK); or

Gastrointestinal System / Excision / Large Intestine (UMLS:ICD10PCS:0DBE); or

Gastrointestinal System / Excision / Cecum (UMLS:ICD10PCS:0DBH); or

Gastrointestinal System / Excision / Descending Colon (UMLS:ICD10PCS:0DBM); or

Gastrointestinal System / Resection / Cecum (UMLS:ICD10PCS:0DTH); or

Gastrointestinal System / Resection / Large Intestine (UMLS:ICD10PCS:0DTE); or

Gastrointestinal System / Resection / Large Intestine, Right (UMLS:ICD10PCS:0DTF); or

Gastrointestinal System / Resection / Ileum (UMLS:ICD10PCS:0DTB); or

Gastrointestinal System / Resection / Sigmoid Colon (UMLS:ICD10PCS:0DTN); or

Gastrointestinal System / Resection / Large Intestine, Left (UMLS:ICD10PCS:0DTG); or

Gastrointestinal System / Resection / Descending Colon (UMLS:ICD10PCS:0DTM); or

Gastrointestinal System / Resection / Ascending Colon (UMLS:ICD10PCS:0DTK); or

Gastrointestinal System / Resection / Transverse Colon (UMLS:ICD10PCS:0DTL); or

Gastrointestinal System / Bypass / Sigmoid Colon (UMLS:ICD10PCS:0D1N); or

Gastrointestinal System / Bypass / Transverse Colon (UMLS:ICD10PCS:0D1L); or

Gastrointestinal System / Bypass / Large Intestine (UMLS:ICD10PCS:0D1E); or

Gastrointestinal System / Bypass / Ascending Colon (UMLS:ICD10PCS:0D1K); or

Gastrointestinal System / Bypass / Descending Colon (UMLS:ICD10PCS:0D1M); or

Gastrointestinal System / Dilation / Large Intestine (UMLS:ICD10PCS:0D7E); or

Gastrointestinal System / Dilation / Ascending Colon (UMLS:ICD10PCS:0D7K); or

Gastrointestinal System / Dilation / Transverse Colon (UMLS:ICD10PCS:0D7L); or

Gastrointestinal System / Dilation / Cecum (UMLS:ICD10PCS:0D7H); or

Gastrointestinal System / Dilation / Sigmoid Colon (UMLS:ICD10PCS:0D7N); or

Laparoscopy, surgical; enterectomy, resection of small intestine, single resection and anastomosis

(UMLS:CPT:44202); or

Laparoscopy, surgical; each additional small intestine resection and anastomosis (List separately in addition to code

for primary procedure) (UMLS:CPT:44203); or

Laparoscopy, surgical; ileostomy or jejunostomy, non-tube (UMLS:CPT:44187); or

Laparoscopy, surgical, closure of enterostomy, large or small intestine, with resection and anastomosis

(UMLS:CPT:44227); or

Enterectomy, resection of small intestine (UMLS:CPT:1007438); or

Intestinal stricturoplasty (enterotomy and enterorrhaphy) with or without dilation, for intestinal obstruction

(UMLS:CPT:44615); or

Closure of intestinal cutaneous fistula (UMLS:CPT:44640); or

Closure of enteroenteric or enterocolic fistula (UMLS:CPT:44650); or

Gastrointestinal System / Excision / Ileum (UMLS:ICD10PCS:0DBB); or

Gastrointestinal System / Excision / Small Intestine (UMLS:ICD10PCS:0DB8); or

Gastrointestinal System / Excision / Jejunum (UMLS:ICD10PCS:0DBA); or

Gastrointestinal System / Resection / Ileum (UMLS:ICD10PCS:0DTB); or

Gastrointestinal System / Resection / Small Intestine (UMLS:ICD10PCS:0DT8); or

Gastrointestinal System / Bypass / Ileum (UMLS:ICD10PCS:0D1B); or

Gastrointestinal System / Bypass / Jejunum (UMLS:ICD10PCS:0D1A); or

Gastrointestinal System / Bypass / Small Intestine (UMLS:ICD10PCS:0D18); or

Gastrointestinal System / Bypass / Duodenum (UMLS:ICD10PCS:0D19); or

Gastrointestinal System / Dilation / Ileum (UMLS:ICD10PCS:0D7B); or

Gastrointestinal System / Dilation / Small Intestine (UMLS:ICD10PCS:0D78); or

Gastrointestinal System / Dilation / Jejunum (UMLS:ICD10PCS:0D7A); or

Gastrointestinal System / Dilation / Ileocecal Valve (UMLS:ICD10PCS:0D7C); or

Gastrointestinal System / Dilation / Duodenum (UMLS:ICD10PCS:0D79); or

Placement of seton (UMLS:CPT:46020); or

Incision and drainage, perianal abscess, superficial (UMLS:CPT:46050); or

Removal of anal seton, other marker (UMLS:CPT:46030); or

Incision and drainage of ischiorectal and/or perirectal abscess (separate procedure) (UMLS:CPT:46040); or

Incision and drainage of ischiorectal or intramural abscess, with fistulectomy or fistulotomy, submuscular, with or

without placement of seton (UMLS:CPT:46060); or

Incision and drainage of intramural, intramuscular, or submucosal abscess, transanal, under anesthesia

(UMLS:CPT:46045); or

Surgical treatment of anal fistula (fistulectomy/fistulotomy) (UMLS:CPT:1007721); or

Closure of anal fistula with rectal advancement flap (UMLS:CPT:46288); or

Fissurectomy, including sphincterotomy, when performed (UMLS:CPT:46200); or

Gastrointestinal System / Excision / Anus (UMLS:ICD10PCS:0DBQ); or

Gastrointestinal System / Excision / Anal Sphincter (UMLS:ICD10PCS:0DBR); or

Gastrointestinal System / Resection / Anus (UMLS:ICD10PCS:0DTQ); or

Gastrointestinal System / Dilation / Anus (UMLS:ICD10PCS:0D7Q); or

Gastrointestinal System / Excision / Sigmoid Colon (UMLS:ICD10PCS:0DBN).

Large Intestine Surgery

**Patients *must* have:**

***any* of the following:**

Laparoscopy, surgical; colectomy, partial, with removal of terminal ileum with ileocolostomy (UMLS:CPT:44205); or

Laparoscopy, surgical; colectomy, total, abdominal, without proctectomy, with ileostomy or ileoproctostomy

(UMLS:CPT:44210); or

Laparoscopy, surgical; colectomy, partial, with anastomosis (UMLS:CPT:44204); or

Laparoscopy, surgical; colectomy, partial, with end colostomy and closure of distal segment (Hartmann type

procedure) (UMLS:CPT:44206); or

Laparoscopy, surgical; colectomy, partial, with anastomosis, with coloproctostomy (low pelvic anastomosis)

(UMLS:CPT:44207); or

Laparoscopy, surgical; colectomy, total, abdominal, with proctectomy, with ileoanal anastomosis, creation of ileal

reservoir (S or J), with loop ileostomy, includes rectal mucosectomy, when performed (UMLS:CPT:44211); or

Colectomy, partial, with removal of terminal ileum with ileocolostomy (UMLS:CPT:44160); or

Colectomy, partial (UMLS:CPT:1007455); or

Colectomy, total, abdominal, without proctectomy (UMLS:CPT:1007463); or

Gastrointestinal System / Excision / Sigmoid Colon (UMLS:ICD10PCS:0DBN); or

Gastrointestinal System / Excision / Large Intestine, Left (UMLS:ICD10PCS:0DBG); or

Gastrointestinal System / Excision / Transverse Colon (UMLS:ICD10PCS:0DBL); or

Gastrointestinal System / Excision / Large Intestine, Right (UMLS:ICD10PCS:0DBF); or

Gastrointestinal System / Excision / Ascending Colon (UMLS:ICD10PCS:0DBK); or

Gastrointestinal System / Excision / Large Intestine (UMLS:ICD10PCS:0DBE); or

Gastrointestinal System / Excision / Cecum (UMLS:ICD10PCS:0DBH); or

Gastrointestinal System / Excision / Descending Colon (UMLS:ICD10PCS:0DBM); or

Gastrointestinal System / Resection / Cecum (UMLS:ICD10PCS:0DTH); or

Gastrointestinal System / Resection / Large Intestine (UMLS:ICD10PCS:0DTE); or

Gastrointestinal System / Resection / Large Intestine, Right (UMLS:ICD10PCS:0DTF); or

Gastrointestinal System / Resection / Sigmoid Colon (UMLS:ICD10PCS:0DTN); or

Gastrointestinal System / Resection / Large Intestine, Left (UMLS:ICD10PCS:0DTG); or

Gastrointestinal System / Resection / Descending Colon (UMLS:ICD10PCS:0DTM); or

Gastrointestinal System / Resection / Ascending Colon (UMLS:ICD10PCS:0DTK); or

Gastrointestinal System / Resection / Transverse Colon (UMLS:ICD10PCS:0DTL); or

Gastrointestinal System / Bypass / Sigmoid Colon (UMLS:ICD10PCS:0D1N); or

Gastrointestinal System / Bypass / Transverse Colon (UMLS:ICD10PCS:0D1L); or

Gastrointestinal System / Bypass / Large Intestine (UMLS:ICD10PCS:0D1E); or

Gastrointestinal System / Bypass / Ascending Colon (UMLS:ICD10PCS:0D1K); or

Gastrointestinal System / Bypass / Descending Colon (UMLS:ICD10PCS:0D1M); or

Gastrointestinal System / Dilation / Large Intestine (UMLS:ICD10PCS:0D7E); or

Gastrointestinal System / Dilation / Ascending Colon (UMLS:ICD10PCS:0D7K); or

Gastrointestinal System / Dilation / Transverse Colon (UMLS:ICD10PCS:0D7L); or

Gastrointestinal System / Dilation / Cecum (UMLS:ICD10PCS:0D7H); or

Gastrointestinal System / Dilation / Sigmoid Colon (UMLS:ICD10PCS:0D7N).

Small Intestine Surgery

**Patients *must* have:**

***any* of the following:**

Laparoscopy, surgical; enterectomy, resection of small intestine, single resection and anastomosis

(UMLS:CPT:44202); or

Laparoscopy, surgical; each additional small intestine resection and anastomosis (List separately in addition to code

for primary procedure) (UMLS:CPT:44203); or

Laparoscopy, surgical; ileostomy or jejunostomy, non-tube (UMLS:CPT:44187); or

Laparoscopy, surgical, closure of enterostomy, large or small intestine, with resection and anastomosis

(UMLS:CPT:44227); or

Enterectomy, resection of small intestine (UMLS:CPT:1007438); or

Intestinal stricturoplasty (enterotomy and enterorrhaphy) with or without dilation, for intestinal obstruction

(UMLS:CPT:44615); or

Closure of intestinal cutaneous fistula (UMLS:CPT:44640); or

Closure of enteroenteric or enterocolic fistula (UMLS:CPT:44650); or

Gastrointestinal System / Excision / Ileum (UMLS:ICD10PCS:0DBB); or

Gastrointestinal System / Excision / Small Intestine (UMLS:ICD10PCS:0DB8); or

Gastrointestinal System / Excision / Jejunum (UMLS:ICD10PCS:0DBA); or

Gastrointestinal System / Resection / Ileum (UMLS:ICD10PCS:0DTB); or

Gastrointestinal System / Resection / Small Intestine (UMLS:ICD10PCS:0DT8); or

Gastrointestinal System / Bypass / Ileum (UMLS:ICD10PCS:0D1B); or

Gastrointestinal System / Bypass / Jejunum (UMLS:ICD10PCS:0D1A); or

Gastrointestinal System / Bypass / Small Intestine (UMLS:ICD10PCS:0D18); or

Gastrointestinal System / Bypass / Duodenum (UMLS:ICD10PCS:0D19); or

Gastrointestinal System / Dilation / Ileum (UMLS:ICD10PCS:0D7B); or

Gastrointestinal System / Dilation / Small Intestine (UMLS:ICD10PCS:0D78); or

Gastrointestinal System / Dilation / Jejunum (UMLS:ICD10PCS:0D7A); or

Gastrointestinal System / Dilation / Ileocecal Valve (UMLS:ICD10PCS:0D7C); or

Gastrointestinal System / Dilation / Duodenum (UMLS:ICD10PCS:0D79).

Anal Surgery

**Patients *must* have:**

***any* of the following:**

Placement of seton (UMLS:CPT:46020); or

Incision and drainage, perianal abscess, superficial (UMLS:CPT:46050); or

Removal of anal seton, other marker (UMLS:CPT:46030); or

Incision and drainage of ischiorectal and/or perirectal abscess (separate procedure) (UMLS:CPT:46040); or

Incision and drainage of ischiorectal or intramural abscess, with fistulectomy or fistulotomy, submuscular, with or

without placement of seton (UMLS:CPT:46060); or

Incision and drainage of intramural, intramuscular, or submucosal abscess, transanal, under anesthesia

(UMLS:CPT:46045); or

Surgical treatment of anal fistula (fistulectomy/fistulotomy) (UMLS:CPT:1007721); or

Closure of anal fistula with rectal advancement flap (UMLS:CPT:46288); or

Fissurectomy, including sphincterotomy, when performed (UMLS:CPT:46200); or

Gastrointestinal System / Excision / Anus (UMLS:ICD10PCS:0DBQ); or

Gastrointestinal System / Excision / Anal Sphincter (UMLS:ICD10PCS:0DBR); or

Gastrointestinal System / Resection / Anus (UMLS:ICD10PCS:0DTQ); or

Gastrointestinal System / Dilation / Anus (UMLS:ICD10PCS:0D7Q).

Intestinal Obstruction

**Patients *must* have:**

***any* of the following:**

Crohn's disease of both small and large intestine with intestinal obstruction (UMLS:ICD10CM:K50.812); or

Crohn's disease of small intestine with intestinal obstruction (UMLS:ICD10CM:K50.012); or

Crohn's disease of large intestine with intestinal obstruction (UMLS:ICD10CM:K50.112); or

Crohn's disease, unspecified, with intestinal obstruction (UMLS:ICD10CM:K50.912).

Intestinal Fistula

**Patients *must* have:**

***any* of the following:**

Crohn's disease of both small and large intestine with fistula (UMLS:ICD10CM:K50.813); or

Crohn's disease of small intestine with fistula (UMLS:ICD10CM:K50.013); or

Crohn's disease of large intestine with fistula (UMLS:ICD10CM:K50.113); or

Crohn's disease, unspecified, with fistula (UMLS:ICD10CM:K50.913).

Overall Complications of Intestine

**Patients *must* have:**

***any* of the following:**

Crohn's disease of both small and large intestine with complications (UMLS:ICD10CM:K50.81); or

Crohn's disease of small intestine with complications (UMLS:ICD10CM:K50.01); or

Crohn's disease of large intestine with complications (UMLS:ICD10CM:K50.11); or

Crohn's disease, unspecified, with complications (UMLS:ICD10CM:K50.91).

CRP ≥10 mg/L

**Patients *must* have:**

C reactive protein [Mass/volume] in Serum, Plasma or Blood (TNX:9063) (at least 10.00 mg/L (most recent
 occurrence)).

Switch to Other Advanced Therapy

**Patients *must* have:**

***any* of the following:**

infliximab (NLM:RXNORM:191831); or

Infliximab (UMLS:CPT:80230); or

Infliximab therapy (UMLS:SNOMED:713517003); or

Injection, infliximab, excludes biosimilar, 10 mg (UMLS:HCPCS:J1745); or

Adalimumab (UMLS:CPT:80145); or

adalimumab (NLM:RXNORM:327361); or

Injection, adalimumab, 20 mg (deprecated 2026) (UMLS:HCPCS:J0135); or

Adalimumab therapy (UMLS:SNOMED:722334000); or

certolizumab pegol (NLM:RXNORM:709271); or

Injection, adalimumab, 1 mg (UMLS:HCPCS:J0139); or

golimumab (NLM:RXNORM:819300); or

Injection, golimumab, 1 mg, for intravenous use (UMLS:HCPCS:J1602); or

vedolizumab (NLM:RXNORM:1538097); or

upadacitinib (NLM:RXNORM:2196092); or

Vedolizumab therapy (UMLS:SNOMED:782712008); or

Vedolizumab (UMLS:CPT:80280); or

Injection, vedolizumab, intravenous, 1 mg (UMLS:HCPCS:J3380); or

Injection, vedolizumab, 1 mg (deprecated 2015) (UMLS:HCPCS:C9026); or

tofacitinib (NLM:RXNORM:1357536).

Venous Thromboembolism

**Patients *must* have:**

***any* of the following:**

Pulmonary embolism (UMLS:ICD10CM:I26); or

Acute embolism and thrombosis of unspecified deep veins of lower extremity (UMLS:ICD10CM:I82.40); or

Acute embolism and thrombosis of deep veins of lower extremity (UMLS:ICD10CM:I82.4).

Major Adverse Cardiovascular Events (MACE)

**Patients *must* have:**

***any* of the following:**

Acute myocardial infarction (UMLS:ICD10CM:I21); or

Cerebral infarction (UMLS:ICD10CM:I63); or

Heart failure (UMLS:ICD10CM:I50); or

Deceased (Deceased).

Opportunistic Infections

**Patients *must* have:**

***any* of the following:**

Zoster [herpes zoster] (UMLS:ICD10CM:B02); or

Toxoplasmosis (UMLS:ICD10CM:B58); or

Tuberculosis (UMLS:ICD10CM:A15-A19); or

Nocardiosis (UMLS:ICD10CM:A43); or

Enterocolitis due to Clostridium difficile (UMLS:ICD10CM:A04.7); or

Legionnaires' disease (UMLS:ICD10CM:A48.1); or

Listeriosis (UMLS:ICD10CM:A32); or

Pneumocystosis (UMLS:ICD10CM:B59); or

Candidiasis (UMLS:ICD10CM:B37); or

Cytomegaloviral disease (UMLS:ICD10CM:B25); or

Aspergillosis (UMLS:ICD10CM:B44).
